# A frame-shift mutation in *COMTD1* is associated with impaired pheomelanin pigmentation in chicken

**DOI:** 10.1371/journal.pgen.1010724

**Published:** 2023-04-17

**Authors:** Huijuan Bi, Jonas Tranell, Dawn C. Harper, Weifeng Lin, Jingyi Li, Anders R. Hellström, Mårten Larsson, Carl-Johan Rubin, Chao Wang, Shumaila Sayyab, Susanne Kerje, Bertrand Bed’hom, David Gourichon, Shosuke Ito, Kazumasa Wakamatsu, Michèle Tixier-Boichard, Michael S. Marks, Daniel Globisch, Leif Andersson

**Affiliations:** 1 Science for Life Laboratory, Department of Medical Biochemistry and Microbiology, Uppsala University, Uppsala, Sweden; 2 Department of Pathology & Laboratory Medicine and Department of Physiology, Children’s Hospital of Philadelphia and University of Pennsylvania Perelman School of Medicine, Philadelphia, United States of America; 3 Department of Chemistry - BMC, Uppsala University, Uppsala, Sweden; 4 Key Laboratory of Agricultural Animal Genetics, Breeding and Reproduction of Ministry of Education, College of Animal Science and Technology, Huazhong Agricultural University, Wuhan, Hubei, China; 5 Department of Animal Breeding and Genetics, Swedish University of Agricultural Sciences, Uppsala, Sweden; 6 Université Paris-Saclay, INRAE, AgroParisTech, GABI, F-78350 Jouy-en-Josas, France; 7 INRAE, PEAT, Nouzilly, France; 8 Institute for Melanin Chemistry, Fujita Health University, Toyoake, Aichi, Japan; 9 Department of Veterinary Integrative Biosciences, Texas A&M University, College Station, United States of America; University of Bern Faculty of Veterinary Medicine: Universitat Bern Vetsuisse Fakultat, SWITZERLAND

## Abstract

The biochemical pathway regulating the synthesis of yellow/red pheomelanin is less well characterized than the synthesis of black/brown eumelanin. Inhibitor of gold (IG phenotype) is a plumage colour variant in chicken that provides an opportunity to further explore this pathway since the recessive allele (*IG*) at this locus is associated with a defect in the production of pheomelanin. *IG/IG* homozygotes display a marked dilution of red pheomelanin pigmentation, whilst black pigmentation (eumelanin) is only slightly affected. Here we show that a 2-base pair insertion (frame-shift mutation) in the 5^th^ exon of the *Catechol-O-methyltransferase containing domain 1* gene (*COMTD1*), expected to cause a complete or partial loss-of-function of the COMTD1 enzyme, shows complete concordance with the IG phenotype within and across breeds. We show that the COMTD1 protein is localized to mitochondria in pigment cells. Knockout of *Comtd1* in a mouse melanocytic cell line results in a reduction in pheomelanin metabolites and significant alterations in metabolites of glutamate/glutathione, riboflavin, and the tricarboxylic acid cycle. Furthermore, *COMTD1* overexpression enhanced cellular proliferation following chemical-induced transfection, a potential inducer of oxidative stress. These observations suggest that COMTD1 plays a protective role for melanocytes against oxidative stress and that this supports their ability to produce pheomelanin.

## Author summary

Vertebrates possess two types of melanin, red/yellow pheomelanin and black/brown eumelanin. In this study, we report that the recessive Inhibitor of gold phenotype in chicken, which causes a severe defect in pheomelanin pigmentation, is associated with a mutation that most likely inactivates the *COMTD1* gene. This gene encodes an *O*-methyltransferase enzyme and is present throughout vertebrate evolution, but is one of the many genes in vertebrate genomes for which the biological function is still poorly understood. This is the first report of a *COMTD1* mutation associated with a phenotypic effect. We show that the COMTD1 protein is present in mitochondria in pigment cells. Furthermore, inactivation of the gene in a mouse pigment cell line results in a significant reduction in metabolites that are important for the synthesis of pheomelanin. We hypothesize that COMTD1 activity protects pigment cells from oxidative stress and that inactivation of this function impairs the production of pheomelanin. It is likely that COMTD1 has a similar function in other cell types. This study establishes this chicken mutation as a model for further studies of COMTD1 function.

## Introduction

Deposition of melanins in skin, hair, or feathers is essential for pigmentation in birds and mammals [1]. Melanins come in two types: black/brown eumelanins and yellow/red pheomelanins. Skin, hair, and feather colour is determined by the amount and ratio of the type of melanin that is deposited [2]. Pigmentation serves many different functions in animals, such as camouflage, mate attraction, and protection against ultraviolet radiation (UVR). The latter is mainly achieved by eumelanins, whereas pheomelanins have phototoxic properties because they generate reactive oxygen species (ROS) upon UVR exposure [3–5].

Melanins are produced within epidermal melanocytes within membrane-bound compartments called melanosomes [6,7]. The rate-limiting reaction in melanogenesis, the oxidation of L-tyrosine to dopaquinone (DQ), is catalysed by tyrosinase. A non-functional tyrosinase leads to disrupted synthesis of melanin and is associated with oculocutaneous albinism type 1 [8]. Two tyrosinase-related proteins, TYRP1 and TYRP2/dopachrome tautomerase (DCT), also contribute to the synthesis of eumelanins. By contrast, pheomelanins are thought to be spontaneously produced from L-tyrosine in the presence of the amino acid cysteine, low levels of tyrosinase, and a lower pH [9–11]. During melanin synthesis, superoxide anion (O_2_^-^) and hydrogen peroxide (H_2_O_2_) are generated at various steps, potentially exposing melanocytes to oxidative stress. Indeed, excessive ROS production from either endogenous or exogenous sources can disrupt melanocyte homeostasis, compromising their survival or leading to their malignant transformation [12,13]. In most cell types, ROS are mainly generated in the oxidative reaction process of mitochondrial respiratory chain as by-products of normal cellular metabolism [14]. Modification of the redox state and increased ROS production within mitochondria have major consequences for both mitochondrial and extramitochondrial processes. Disrupted cell proliferation is one of the consequences of mitochondrial stress, as mitochondria are the primary source of energy production for fundamental cellular reactions [15,16]. In addition, accumulated ROS resulting from mitochondrial stress can cause DNA damage and disrupt signalling pathways that regulate cell proliferation [17,18]. Additionally, mitochondrial fission activity is associated with cell proliferation [19].

**Fig 1:**
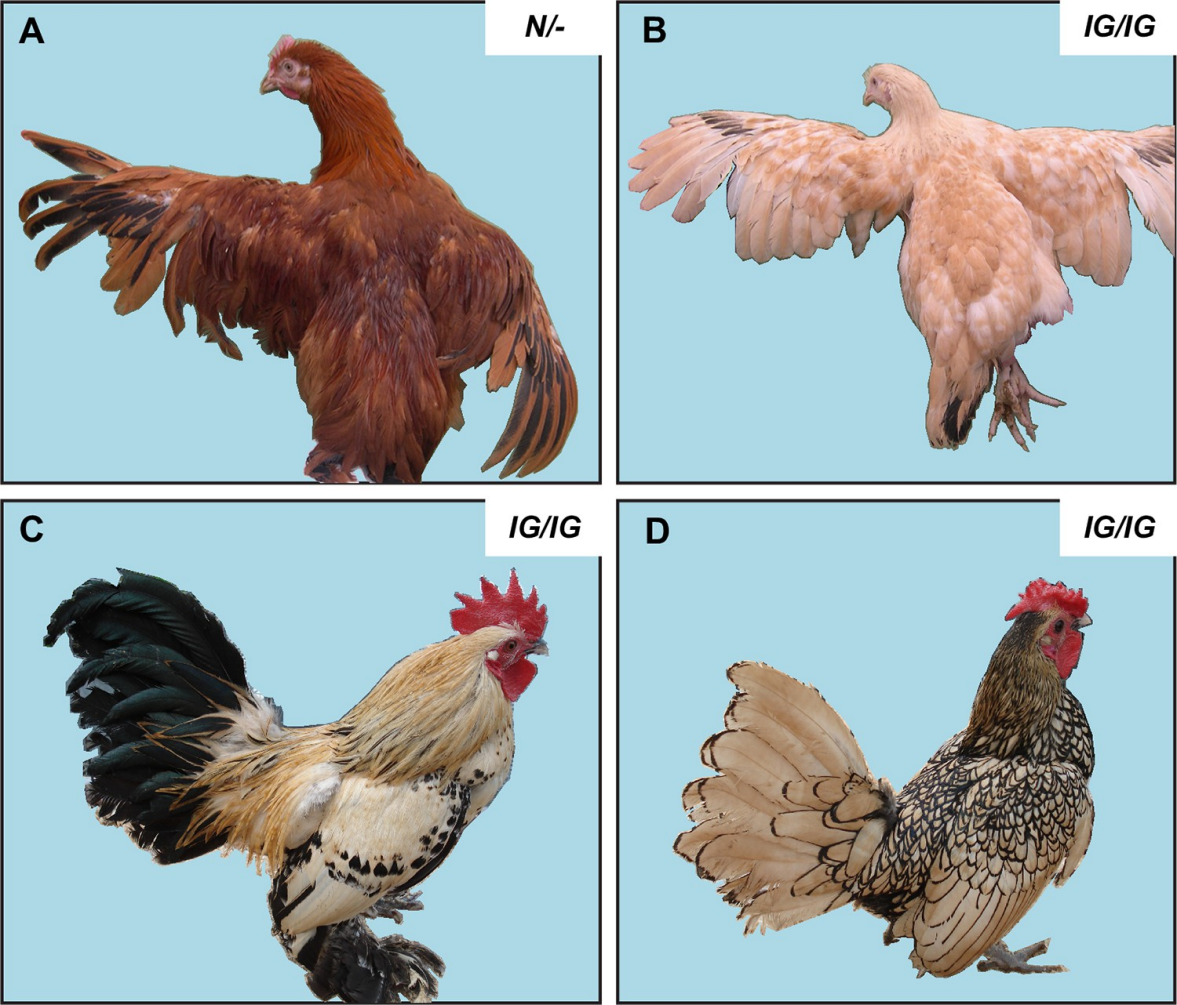
Illustration of plumage phenotypes associated with different genotypes at the *Inhibitor of gold* locus in chicken on different genetic backgrounds. The birds in (A) and (B) carry the bottom recessive wheaten allele (*Y*) at the *MC1R* locus and shows red pheomelanin-based pigmentation. The birds in (C) and (D) carry the brown allele (*B*) at the same locus that allows expression of both eumelanin and pheomelanin and IG dilution is apparent as regards pheomelanin pigmentation. (A) and (B) depict F_2_ birds from the mapping pedigree with the wild-type phenotype or the recessive IG phenotype (*IG/IG*), respectively. (C) and (D) depict two *IG/IG* birds from the Lemon Millefleur Sabelpoot (**Fig 1C**) and Sebright-Lemon (**Fig 1D**) breeds, respectively. Photo by Michèle Tixier-Boichard (A and B) and C and D were taken by Nicolas Bruneau, INRAE.

While the mouse is the most widely-used mammalian model organism for understanding the genetic basis of pigment variation (http://www.espcr.org/micemut/), the chicken is the prime avian model organism, and the immense phenotypic variation in plumage and skin colour within and between chicken breeds has yielded numerous genetic insights into the regulation of pigmentation [20]. The Inhibitor of gold (IG) or Cream phenotype was first described by Taylor [21] and later analysed by Punnett [22]. The autosomal recessive *IG* allele mainly dilutes the red/pheomelanic components of the plumage with minor effects on black/eumelanic components (**Fig 1**). A general dilution of pigmentation is present in homozygous *IG/IG* birds that are also homozygous for the recessive wheaten allele (*Y*) at the *MC1R* locus and therefore exhibit only red pheomelanin pigmentation (**Fig 1B**). The phenotype is apparent already at hatch. In contrast, IG birds carrying other *MC1R* alleles that allow expression of both eumelanin and pheomelanin show a dilution of red pheomelanic pigmentation but no visible dilution of black eumelanic pigmentation (**Figs 1C and 1D**). Male IG chickens display a greater dilution of pheomelanin pigmentation than IG females, suggesting the involvement of sex-specific factors. The *IG* allele is fairly widespread among chicken breeds around the world including some White Leghorn lines [23], some broiler lines, Lemon Millefleur Sabelpoot (**Fig 1C**), Sebright-Lemon (**Fig 1D**), and Lemon Spangled Hamburg. However, the mutated gene responsible for the IG phenotype has not yet been identified.

In the present study we present genetic data demonstrating that the IG phenotype shows complete concordance with a frame-shift mutation in the *catechol-O-methyltransferase containing domain 1* gene (*COMTD1*). COMTD1 is associated with mitochondrial fractions of several cell types [24]. *COMTD1* is ubiquitously expressed in human and mouse [25] but is expressed most highly in gall bladder, small intestine, parathyroid gland, and renal tubes of the kidney (http://www.proteinatlas.org/ENSG00000165644). The crystal structure of the fragment of human COMTD1 lacking its N-terminal transmembrane domain (Protein Database accession number: 2AVD) suggests that it forms a dimer and binds the co-factor *S*-adenosyl-methionine (SAM). COMTD1 is predicted to contain an *O*-methyltransferase domain and shows strong sequence similarity with the well-characterized catechol-*O*-methyltransferase (COMT). COMT is an *O*-methyltransferase that catalyzes the transfer of a methyl group from SAM [26] to a wide number of catechol-substrates including neurotransmitters [27], catechol estrogens [28], ascorbic acid [29], catecholic xenobiotics [30], and dihydroxyindole intermediates of melanin [31]. *Comt*-deficient mice show dramatic shifts in catecholamine metabolites [32–34]. COMT has been reported to play a role in oxidative stress pathways, mainly through the regulation of ROS levels. Knockdown of *COMT* expression in human endometrial glandular cells increased the propensity of estradiol or catecholestrogens to induce ROS [35]. Inadequate COMT activity in substantia nigra pars compacta results in increased dopamine and its catechol-containing metabolites, which consequently lead to oxidative damage to neuronal cells [36]. COMT has been suggested to protect melanocytes from the toxic indole intermediates produced during melanogenesis [37–43], and *in vitro* COMT catalyzes *O*-methylation of the melanin substrate L-Dopa to 3-*O*-methoxytyrosine [44]. Our data suggest that *COMTD1* might also regulate melanogenesis, but that its function is limited to mitochondria and not to melanosomes in which L-Dopa is generated. Moreover, we document a complex interplay between melanogenesis and cellular metabolism, suggesting that both *COMT* and *COMTD1* protect melanocytes from oxidative stress.

## Results

### Minor deviation from expected Mendelian segregation

A three-generation intercross was set up between three Rhode Island Red (RIR) birds carrying the wild-type allele at the *IG* locus (*N*) and four individuals from an experimental population fixed for the *Inhibitor of Gold* allele to produce a pedigree for mapping the *IG* locus. A total of 413 F_2_ chickens were born, of which 83 (20.1%) displayed the dilution of pheomelanin pigmentation caused by *IG* homozygosity. This represents a minor but significant deviation from the 25% expected based on Mendelian segregation (χ^2^ = 5.30, df = 1, *P* = 0.02).

### Chemical characterization of feather melanin

**Fig 2:**
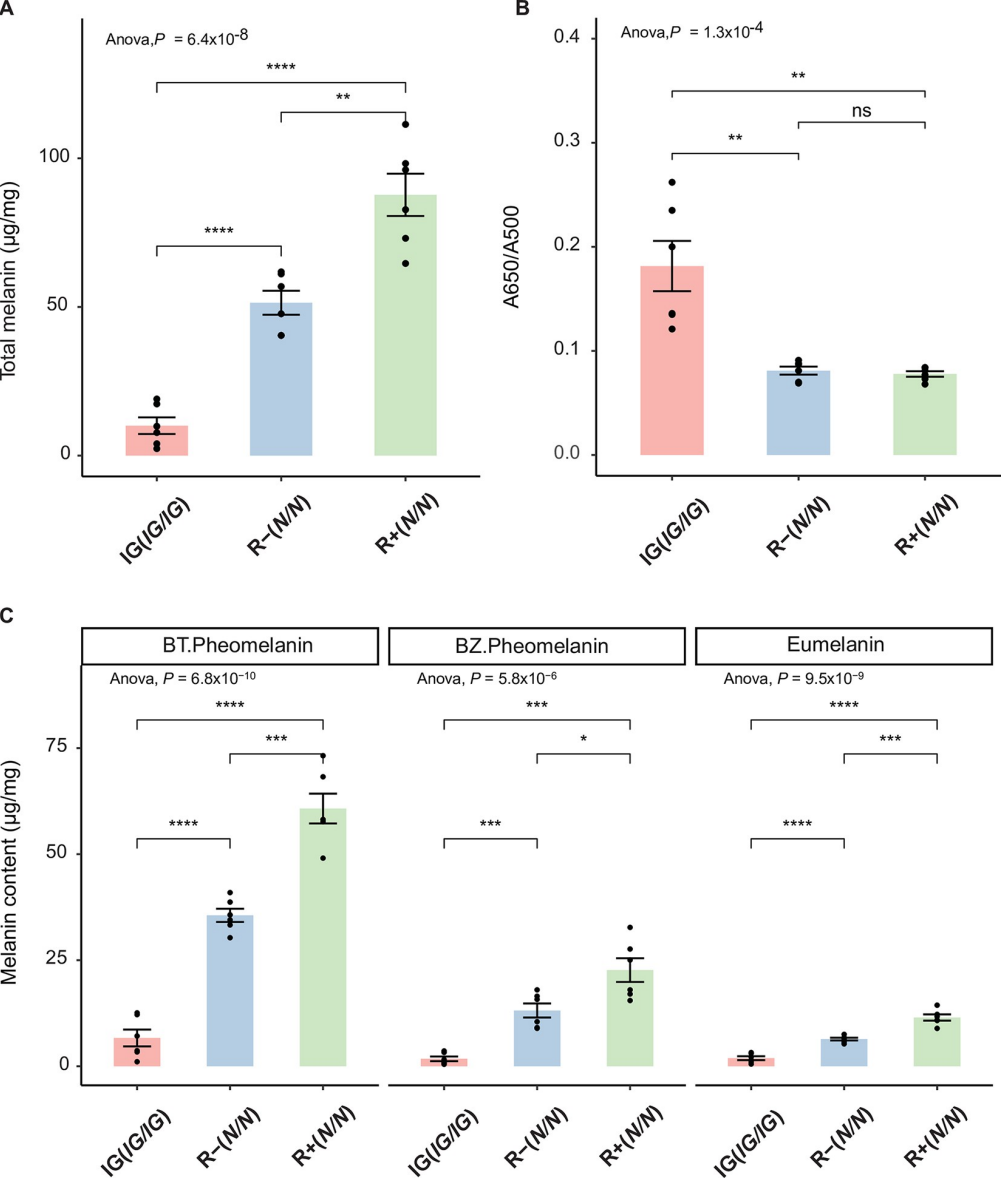
Chemical characterization of feather melanin. (A) depicts levels of total melanin in wild-type birds (R+ and R-) and in IG birds analyzed by Soluene-350 solubilization. (B) depicts A650/A500 ratios analyzed by Soluene-350 solubilization. (C) depicts eumelanin (EM), benzothiazine-pheomelanin (BT-PM), and benzothiazole-pheomelanin (BZ-PM) analyzed as PTCA, 4-AHP, and TTCA, respectively. Feather samples were obtained from neck regions from 3 males and 3 females. Results are shown with the means ± SEM of 6 birds. ns: not significant, P > 0.05; *: P < 0.05; **: P <0.01; ***: P <0.001; ****: P <0.0001 (Student’s t test).

The melanin content in feathers was analyzed in RIR (homozygous wild-type, *N/N)* and IG (*IG*/*IG*) birds. All birds show the recessive wheaten phenotype (MC1R*Y/Y) and produce predominantly red pheomelanin (**Fig 1A and 1B**). The RIR birds came from two different lines (R+ and R-) that were divergently selected for feed efficiency and that exhibit more and less intensive pheomelanin pigmentation, respectively. Solubilized feathers from the neck of both sexes were analyzed for melanin content by spectrophotometry for absorbance at 500 nm (A500) and 650 nm (A650). The A500 value reflects the total amount of eumelanin and pheomelanin combined (total melanin) while the A650/A500 ratio provides a rough estimate of the ratio of eumelanin to pheomelanin–the lower the ratio, the higher the pheomelanin content [45]. In IG birds, the level of total melanin was reduced significantly to about 20% that of R- birds and 10% that of R+ birds (**Fig 2A**). The A650/A500 ratio indicates that IG birds show a higher proportion of eumelanin pigmentation compared to the high pheomelanin content of R- and R+ birds (**Fig 2B**), consistent with the severely reduced pheomelanin pigmentation in the IG birds. To better quantify pheomelanin and eumelanin content, products of chemical degradations were analyzed by high-performance liquid chromatography (HPLC). Homogenized feathers in water were subjected to alkaline hydrogen peroxide oxidation (AHPO) [46] and hydroiodic acid (HI) hydrolysis [47]; AHPO generates pyrrole-2,3,5-tricarboxylic acid (PTCA) from eumelanin and thiazole-2,4,5-tricarboxylic acid (TTCA) from benzothiazole (BZ)-type pheomelanin [46], and HI hydrolysis generates 4-amino-3-hydroxyphenylalanine (4-AHP) from benzothiazine (BT)-type pheomelanin. AHPO and HI analysis showed that in IG birds, the levels of eumelanin, BT pheomelanin, and BZ pheomelanin were all reduced significantly to 15%, 20%, and 20% compared to R- birds, respectively (**Fig 2C**). Total melanin values (eumelanin and pheomelanin combined) by HPLC and by spectrophotometry corresponded well. Taken together, these results show that feather content of total melanin and particularly pheomelanin is strongly diminished in IG birds as compared to R- birds and even more strongly as compared to R+ birds.

We also compared pigmentation between R+ and R- birds from the Rhode Island Red breed. R+ birds produced nearly two-fold more pheomelanin, both BT-type and BZ-type, as compared to R- birds (P<0.001; **Fig 2C**). Variations in melanin content were noted between males and females in the three subgroups of birds (R+, R-, and IG), with females showing higher values (**S1 Fig**).

### Linkage analysis

To identify the genetic region responsible for the IG phenotype, linkage analysis was performed using 159 F_2_ individuals from a three-generation intercross segregating at the *IG*-locus. Close linkage between *IG* and the genetic marker MCW250 on chicken chromosome 6 at nucleotide position 16,581,142 bp was identified (LOD score = 39.3; θ = 0.1%). The marker AP3UP3_5Mb, located approximately 3.7 Mb away from MCW250, showed complete linkage with *IG* (LOD score = 49.1; θ = 0.0%; **Table 1**). Addition of more markers in the proximity of AP3UP3_5Mb allowed us to assign the *IG* locus to a 3.71 Mb region on chicken chromosome 6 defined by the closest flanking markers IG1250Kb and AP3UP500Kb, which showed recombination with the *IG* locus (**Table 1**). Further addition of genetic markers within the 3.71 Mb region revealed complete linkage between these markers and *IG*. This was a much larger region lacking recombination events than expected given the number of informative meioses in this study and the average recombination rate on chicken chromosome 6, which has been estimated to be around 3.0 cM/Mb [48]. However, a low rate of recombination in this chromosomal region is also evident from the consensus map for chicken [48]. Thus, the low rate of recombination in the present study does not imply that the *IG* allele may be associated with a chromosomal rearrangement, e.g., an inversion, which suppresses recombination.

**Table 1:** Pair-wise linkage analysis between the *IG* locus and genetic markers on chicken chromosome 6.

Marker	Position in assembly (bp)^a^	Recombination fraction	LOD-score versus *IG*	Position in linkage map (cM)
**IG6**	7,568,812	0.07	31.0	0
**IG5**	9,725,625	0.04	24.8	3.5
**IG1250Kb**	12,025,177	0.01	43.2	7.5
**AP3UP3_5Mb**	12,457,510	0	49.1	8.8
**APUP1500Kb**	14,715,262	0	37.6	9.2
**COMTD1**	15,675,523	0	47.9	9.7
**AP3UP500Kb**	15,733,952	0.01	46.4	11.0
**AP3DWN3.5Mb**	18,351,115	0.03	35.3	14.4
**ADL142**	30,711,168	0.36	2.2	55.3

^a^According to the galGal6 genome assembly.

### Identical-by-Descent mapping

**Fig 3:**
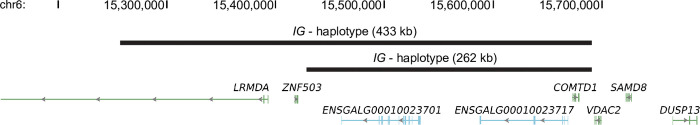
Gene content of the IG interval. The annotation is based on the chicken genome assembly as presented on the UCSC sequence browser. Both the larger (433 kb) and the smaller (262 kb) IBD regions associated with the *IG* allele are marked.

We used an Identical-by-Descent (IBD) mapping approach to refine the localization of the *IG* locus, under the assumption that most, if not all, *IG* chromosomes trace back to one common ancestor. We partially re-sequenced the associated 3.71 Mb region in six birds, representing five different IG populations, one of which was an F_2_ *IG/IG* homozygote from the mapping-population. Combining these data with previously published sequence data from birds not carrying the *IG* allele (red junglefowl and Rhode Island Red) revealed a 432,231 bp minimum shared haplotype (between nucleotide position 15,257,589 and 15,689,820**)** among *IG* chromosomes (**Table 2**). An even smaller IBD-region (261,682 bp, between 15,428,138 and 15, 689,820 bp) was supported by one recombinant chromosome found in one Lemon Spangled Hamburg bird. The larger, high confidence, minimum shared region harboured three ENSEMBL protein-coding gene annotations and two lncRNAs (**Fig 3**). The three protein-coding genes displayed a high sequence similarity to genes in other vertebrates: *LRMDA*, *ZNF503*, and *COMTD1* (Refseq gene predictions) and were thus considered to be true protein-coding genes. The smaller 261,682 bp IBD region contained only one of these protein-coding genes, *COMTD1*, and the two lncRNAs.

**Table 2:** Genotype data at SNP positions of the *IG* locus used for identical-by-descent mapping.

Breed		Ig line (INRA)	Lemon Sebright	Lemon Spangled Hamburg	Lemon Millefleur Sabelpoot	F2 sample from mapping	Ig line (SASSO)	Genome assembly rjf	Rhode Island Red (Pool)^2^	Red jungle fowls (Pool)^2^
n Phenotype		1	1	1	1	1	1	8	8	8
		IG	IG	IG	IG	IG	IG	Red	Red	Red
Nucleotide position^1^	15,182,332	C	Y	.	.	.	.	T	T	T
	15,250,666	T	A	A	A	.	N	.	A	A
	15,250,683	G	.	C	.	.	N	.	.	.
	15,250,703	G	A	A	A	.	N	.	A	A
	15,257,589	wt	het	.	del	.	?	.	.	?
	15,428,090	A	.	T	.	.	.	.	T	N
	15,428,120	C	.	T	.	.	.	.	T	Y
	15,428,138	T	.	G	.	.	.	G	G	N
	15,672,892	C	.	.	N	.	.	A	A	M
	15,673,123	T	.	.	.	.	.	.	K	G
	15,675,283	G	.	.	.	.	.	A	A	A
	15,675,328	T	.	.	.	.	.	C	C	C
	15,675,331	G	.	.	.	.	.	C	C	C
	15,675,404	T	.	.	.	.	.	C	Y	C
	15,675,419	C	.	.	.	.	.	G	G	G
	15,689,820	A	W	.	.	W	.	T	T	W
	15,690,118	G	A	.	.	R	.	A	R	A
	15,714,990	A	R	.	.	R	.	G	G	G

^1^A dot (.) represents the same sequence variant as the INRA IG-line and the nucleotide positions are according to galGal6 genome assembly. Heterozygous positions are coded in accordance with IUPAC nomenclature rules for degenerate bases. A question mark (?) refers to undetermined indel-genotypes. n = number of individuals.

^2^Genotype data retrieved from Rubin *et al*. [91]

### Detection of a candidate causative mutation

To find candidate coding sequence mutations that might result in a loss-of-function responsible for the IG phenotype within the relatively large IBD region associated with *IG*, we performed whole genome sequencing of an *IG/IG* homozygote. A 300 bp fragment library was generated and short paired-end reads (100 bp each) were mapped to the chicken galGal6 genome assembly, giving on average 30X coverage. Large structural variants and polymorphic sites, including single nucleotide polymorphisms (SNPs), insertions, and deletions (InDels), were identified in the high confidence minimum shared *IG* region in comparison to the red junglefowl reference sequence. The causative mutation was assumed to be in the homozygous state in these data due to the recessive mode of inheritance of the IG phenotype. Polymorphisms present in the heterozygous state were excluded from the analyses.

All detected polymorphisms were intersected with the three ENSEMBL gene predictions in the *IG* minimum shared haplotype (**S1 Table**). We also determined the allele frequency of all detected sequence variants among 46 previously sequenced birds all assumed to be wild-type (*N/-*) at the *IG* locus. There were only two variants that were not found in the homozygous state in non-IG birds: a synonymous substitution in *LRMDA* and a 2-bp insertion of CT at nucleotide position 15,675,521 bp in exon 5 of *COMTD1*. The *COMTD1* insertion is predicted to cause a frame shift, and was thus considered to be the strongest candidate mutation to explain the *IG* allele due to its predicted severity. The synonymous substitution in *LRMDA* (chr6: 14,861,168 bp) has a negative conservation score (-4.2) based on the 77 vertebrate PhastCons Conserved Elements Sequences, suggesting that it is not in a conserved site and thus may not be functionally important.

### Confirmation of the association between the 2-bp insertion in *COMTD1* and the IG phenotype

The 2-bp insertion (*CT* allele) was genotyped in a large pedigree of an intercross between a White Leghorn line (Obese Line) and red junglefowl, which segregated at the *IG* and *sex-linked Silver* loci. Sex-linked Silver birds were excluded from the analysis through genotyping of the putative *Silver* mutation in White Leghorns (S*S_WL) [49]. There was a highly significant correlation between the *CT/CT* genotype and dilution of pheomelanin (χ^2^ = 38.6, df = 1, *P* = 5.2x 10^−10^; **S2 Table**). None of the 25 *CT/CT* homozygotes displayed a red plumage while 58 (70.7%) of the 82 birds in the other two genotype classes did. The lack of red plumage in the 29.3% of birds that were not *CT/CT* likely reflects allelic variation at other loci, such as *recessive white*, *champagne*, *lavender*, and/or *Dominant white*, which all dilute pheomelanin pigmentation. Thus, these data support the contention that the 2-bp insertion in *COMTD1* is causative for the IG phenotype.

The 2-bp insertion in *COMTD1* was also genotyped in (*i*) IG birds from five different populations and (*ii*) wild-type birds representing 7 domestic breeds and three junglefowl species all displaying red plumage phenotypes. This screen revealed a complete concordance between homozygosity for the 2-bp insertion (*CT/CT*) and the IG phenotype (**Table 3**). This highly significant association across breeds and complete fixation within breeds for the IG phenotype confirmed that the allele present among this sample of IG birds must have been inherited from a common ancestor. The presence of the *CT*-allele at a low frequency in the Marans population, showing red plumage, does not rule out this mutation as causative since we cannot exclude the possibility that the recessive *IG* allele occurs at a low frequency in this breed. One of the SASSO birds expected to show the IG phenotype was heterozygous for the wild-type allele. This could be explained by a rare phenotype misclassification since alleles at other loci may also dilute pigmentation, or to the presence of genetic heterogeneity at this locus such that other *COMTD1* mutations with a very similar phenotypic effect may occur in this breed.

**Table 3:** Genotype distribution of the 2-bp insertion in *COMTD1* associated with the Inhibitor of Gold (IG) phenotype in different populations sorted by phenotype.

				Genotype		
Breed	Phenotype	n1	n2	*–/–*	*–/CT*	*CT/CT*
IG line (INRA)	IG	1	5	0	0	5
IG line (SASSO)	IG	1	33	0	1	32
Lemon Sebright	IG	1	1	0	0	1
Lemon Spangled Hamburg	IG	1	1	0	0	1
Lemon Millefleur Sabelpoot	IG	1	1	0	0	1
Rhode Island Red	Red	3	20	20	0	0
Czech Golden Pencilled	Red	1	4	4	0	0
Red Villafranquina	Red	1	5	5	0	0
Owl-bearded	Red	1	4	4	0	0
Sicilienne	Red	1	10	10	0	0
Marans	Red	1	10	8	2	0
Smyth Line/Brown Line	Red	1	10	10	0	0
Red junglefowl *(G*. *gallus*)	Red	3	23	23	0	0
Ceylon junglefowl *(G*. *lafayetti*)	Red	1	1	1	0	0
Grey junglefowl (*G*. *sonneratti*)	Red	2	5	5	0	0

n1 = number of populations; n2 = total number of individuals.

We also searched for the presence of the 2-bp insertion in *COMTD1* using publicly available chicken whole genome sequencing (WGS) data, representing 20 different populations of non-IG birds (displaying red plumage phenotypes), and 28 populations of other plumage colours (**S3 and S4 Tables**). Among the 20 non-IG populations, all individuals were homozygous for the wild-type allele except one red junglefowl and one Red Porcelain Booted Bantam which were both heterozygous for the 2-bp insertion. This observation does not rule out this mutation as being causative since the phenotype is a recessive trait. In addition, the *COMTD1* 2-bp insertion mutation was present at a high frequency in White Leghorns (**S4 Table**), and may contribute to the pure white phenotype in this breed. This is possible because it is well known that the *PMEL* mutation underlying dominant white colour in this breed effectively inhibits expression of black eumelanin but is less effective in inhibiting the production of red pheomelanin [50]. One black Java was homozygous for the 2-bp insertion (**S4 Table**), but since black Javas have an entirely eumelanic plumage we do not expect the effect of the *IG* mutation to be noted without measuring melanin content.

### RT-PCR analysis reveals two alternative *COMTD1* transcripts associated with *IG*

**Fig 4:**
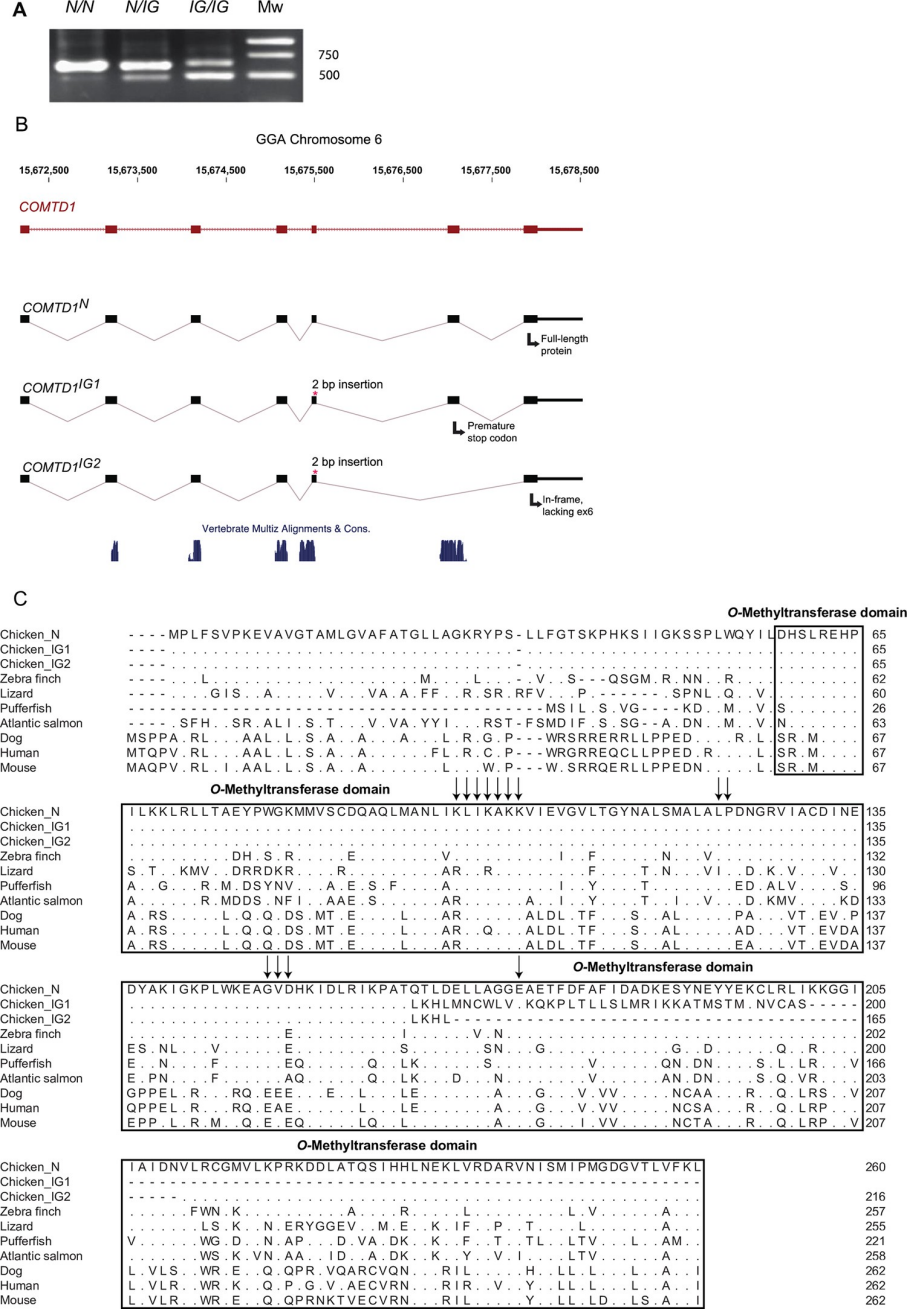
RT-PCR analysis of *COMTD1* using mRNA from feather follicles and protein sequence alignment of COMTD1 homologs. (A) Agarose gel electrophoresis of *COMTD1* RT-PCR products from feather follicles representing all three possible *Ig* genotypes. The *wild-type (N)* allele expresses only the full-length transcript while two transcripts is expressed from the *IG* allele. (B) The annotated *COMTD1* gene with the wild-type transcript (COMTD1^N^) together with the two COMTD1 transcripts (COMTD1^IG1^ and COMTD1^IG2^) transcribed from the *IG*-allele. The bottom track shows vertebrate sequence conservation scores from the USCS browser. (C) Clustal X alignment of predicted COMTD1 sequence in chicken (*Gallus gallus*)–including the putative protein products of COMTD1^IG1^ and COMTD1^IG2^ and homologs in zebra finch (*Taeniopygia guttata*), anolis lizard (*Anolis carolinensis*), green pufferfish (*Tetraodon nigroviridis)*, dog (*Canis familiaris*), mouse (*Mus musculus*), human (*Homo sapiens*) and salmon (*Salmo salar*). Dots (.) indicate identity to the full-length chicken master sequence. The arrows indicate amino acids that are in direct contact with the cofactor, SAM, based on the crystal structure model of COMTD1 (Protein Database accession number: 2AVD). The black square indicates the putative *O*-methyltransferase domain.

RT-PCR analysis of RNA derived from feather follicles representing the three possible genotypes at the *IG* locus revealed that there are two COMTD1 transcripts associated with the *IG* allele. A larger transcript of 3,042 bp, referred to as COMTD1^IG1^, is similar in size to the wild-type COMTD1^N^ transcript (3,040 bp) that encodes the full-length protein. A second transcript of 2,908 bp (COMTD1^IG2^) is ~100 bp smaller than COMTD1^IG1^ and COMTD1^N^ (**Fig 4A**). Sequencing showed that both COMTD1^IG1^ and COMTD1^IG2^ contained the 2-bp insertion but that COMTD1^IG2^ lacks exon 6 and is thus 134 bp smaller than COMTD1^IG1^ (**Fig 4B**).

### IG is associated with a predicted loss of function of *COMTD1*

**Fig 4C** displays the alignment of the COMTD1 protein sequences from eight distantly related organisms including *Gallus gallus* wild-type *COMTD1* and predicted COMTD1^IG1^ and COMTD1^IG2^ (denoted as Chicken_IG1 and Chicken_IG2, respectively). The *Gallus gallus* wild-type COMTD1^N^ protein consists of 260 amino acids with a *C*-terminal *Methyltransf_3 O*-methyltransferase domain (based on the NCBI Conserved Domains Database; E-value: 1.7x10^-79^; pfam:01596). The protein encoded by the COMTD1^IG1^ transcript deviates from COMTD1^N^ at codon 163 and contains a premature stop at codon 201. Thus, the protein encoded by COMTD1^IG1^ lacks the C-terminal 98 amino acids present in the predicted wild-type protein, and these are replaced by 38 amino acids due the frameshift. The protein sequence derived from the COMTD1^IG2^ transcript aligned well to wild-type protein sequence except for the 4 out-of-frame amino acids derived from exon 5 and the 44 missing amino acids encoded by exon 6. As indicated in **Fig 4C**, the mutant isoforms lack considerable portions of the *O*-methyltransferase domain and are therefore predicted to be non-functional.

### COMTD1 localizes to mitochondria in melanocytes

**Fig 5:**
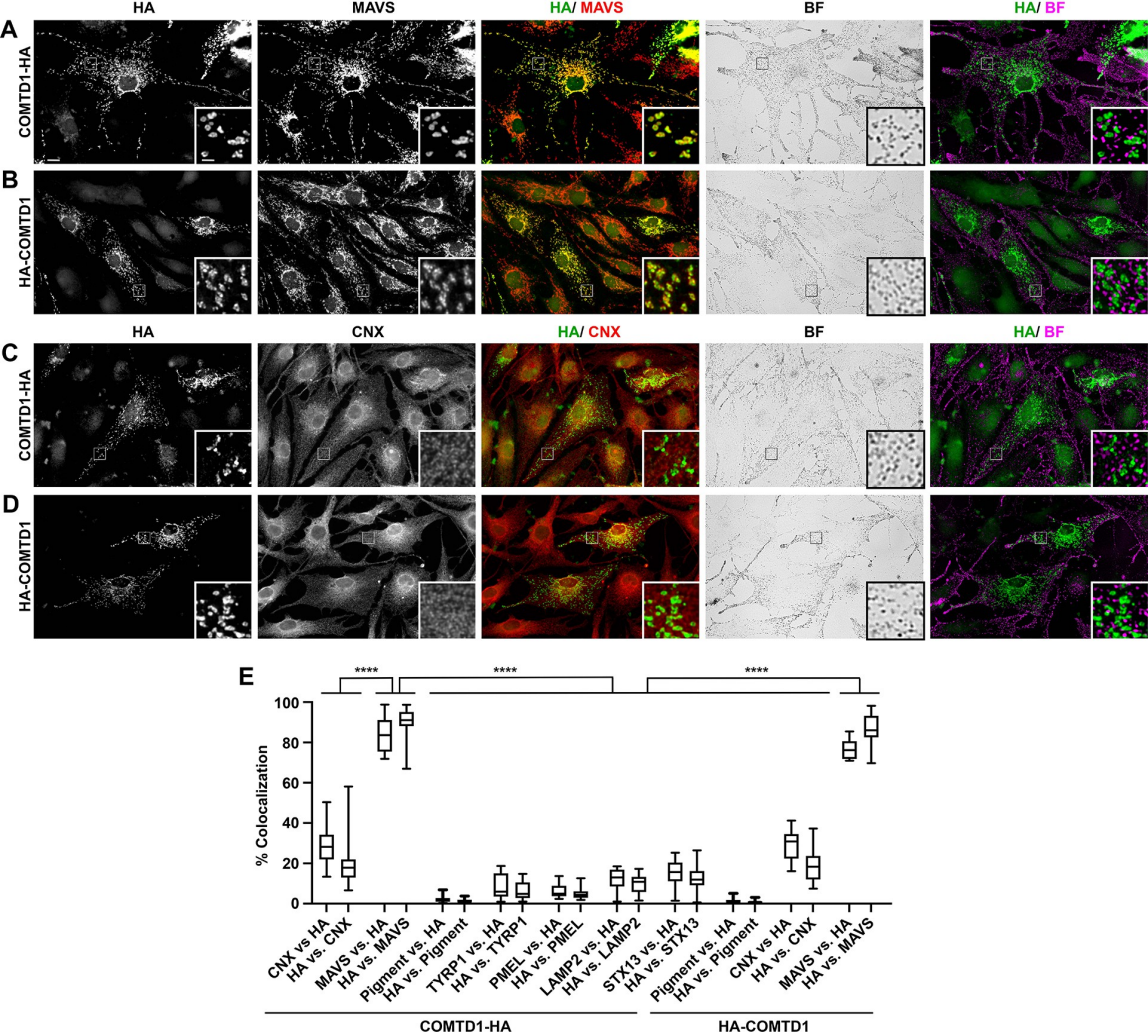
HA-tagged COMTD1 localizes to mitochondria in immortalized mouse melanocytes. (A-D) Immortalized melan-Ink4a cells from Ink4a-deficient C57BL/6J mice were transiently transfected to express COMTD1 fused with the HA11 epitope at either the N-terminus (HA-COMTD1; A, C) or C-terminus (COMTD1-HA; B, D). Two days later, cells were fixed and analyzed by bright field (BF) and immunofluorescence microscopy for HA and either the mitochondrial resident protein MAVS (A, B) or the ER resident protein calnexin (CNX; C, D). Individual images of labelled cells or the bright field image are shown in addition to an overlay of HA (green) with MAVS (red; HA/ MAVS), CNX (red; HA/ CNX), or the pseudocolored bright field image (magenta; HA/BF). Insets show a 5-fold magnified image of the boxed region to emphasize overlap or lack thereof. Main scale bar, 10 μm; inset scale bar, 2 μm. (E) Quantification of the degree of overlap of COMTD1-HA or HA-COMTD1, as indicated, with markers of the ER (CNX; N = 29 for COMTD1-HA, N = 17 for HA-COMTD1), mitochondria (MAVS; N = 25 for COMTD1-HA, N = 16 for HA-COMTD1), mature melanosomes (TYRP1; N = 16), immature melanosomes (PMEL; N = 17), late endosomes/ lysosomes (LAMP2; N = 15), or early endosomes (STX13; N = 21). Data from 4–5 individual experiments are presented as a box and whiskers plot in which the area of overlap is shown relative to the total area occupied by HA (e.g., CNX vs. HA) or by the indicated marker (e.g., HA vs. CNX). See **S2** Fig for examples of the data for TYRP1, PMEL, LAMP2 and STX13. Statistical significance was determined by ordinary one-way ANOVA with Tukey’s tests for multiple comparisons; ****, P < 0.0001.

To define the intracellular localization of COMTD1 in melanocytes, human COMTD1 was fused to the HA11 epitope at either the *N*-terminus (to generate HA-COMTD1) or the *C*-terminus (to generate COMTD1-HA), and the tagged proteins were expressed by transient transfection in melan-Ink4a melanocytes, a highly pigmented immortalized melanocyte cell line derived from C57BL/6J-*Arf*-*Ink4a*^-/-^ mice. Cells were then analyzed by bright field microscopy (to detect pigment granules) and immunofluorescence microscopy for the HA-tagged proteins and markers of multiple compartments (**Figs 5** and **S2)**. Labelling for the HA epitope fused to COMTD1 at either the *N*-terminus or *C*-terminus failed to overlap significantly with pigment granules (1.5 ± 1.3% and 2.2 ± 1.8%, respectively; **Fig 5**) or with markers of mature melanosomes (TYRP1; 8.8 ± 6.3% for COMTD1-HA) or immature melanosomes (PMEL; 6.6 ± 3.8% for COMTD1-HA) (**Figs** 5E, **S2A**, **S2B**, **S2E** and **S2F**), indicating that COMTD1 is unlikely to localize to melanosomes. Moreover, COMTD1 labelling failed to overlap substantially with labelling for markers of the endoplasmic reticulum (calnexin; 29.3 ± 7.5% for HA-COMTD1, 28.5 ± 8.8% for COMTD1-HA)(**Fig 5C**–**5E**) late endosomes/ lysosomes (LAMP2; 12.1 ± 5.4% for COMTD1-HA; **Figs 5E, S2C, and S2G**), or early endosomes (syntaxin 13; 15.0 ± 6.3% for COMTD1-HA; **Figs 5E and S2D**, **S2H**)–all compartments that contribute to assembly, processing, and trafficking of melanosomal proteins [6]. However, COMTD1, fused to HA at either the *N*- or *C*-terminus, overlapped extensively with labelling for the mitochondrial outer membrane protein, MAVS (76.8 ± 5.0% for HA-COMTD1, 83.4 ± 8.7% for COMTD1-HA; **Fig 5A**, 5**B** and 5**E**). These data strongly suggest that COMTD1 localizes predominantly, if not exclusively, to mitochondria, consistent with a tissue-wide mouse mitochondrial proteomics study [24].

### *COMTD1* inactivation alters several metabolic pathways

**Fig 6:**
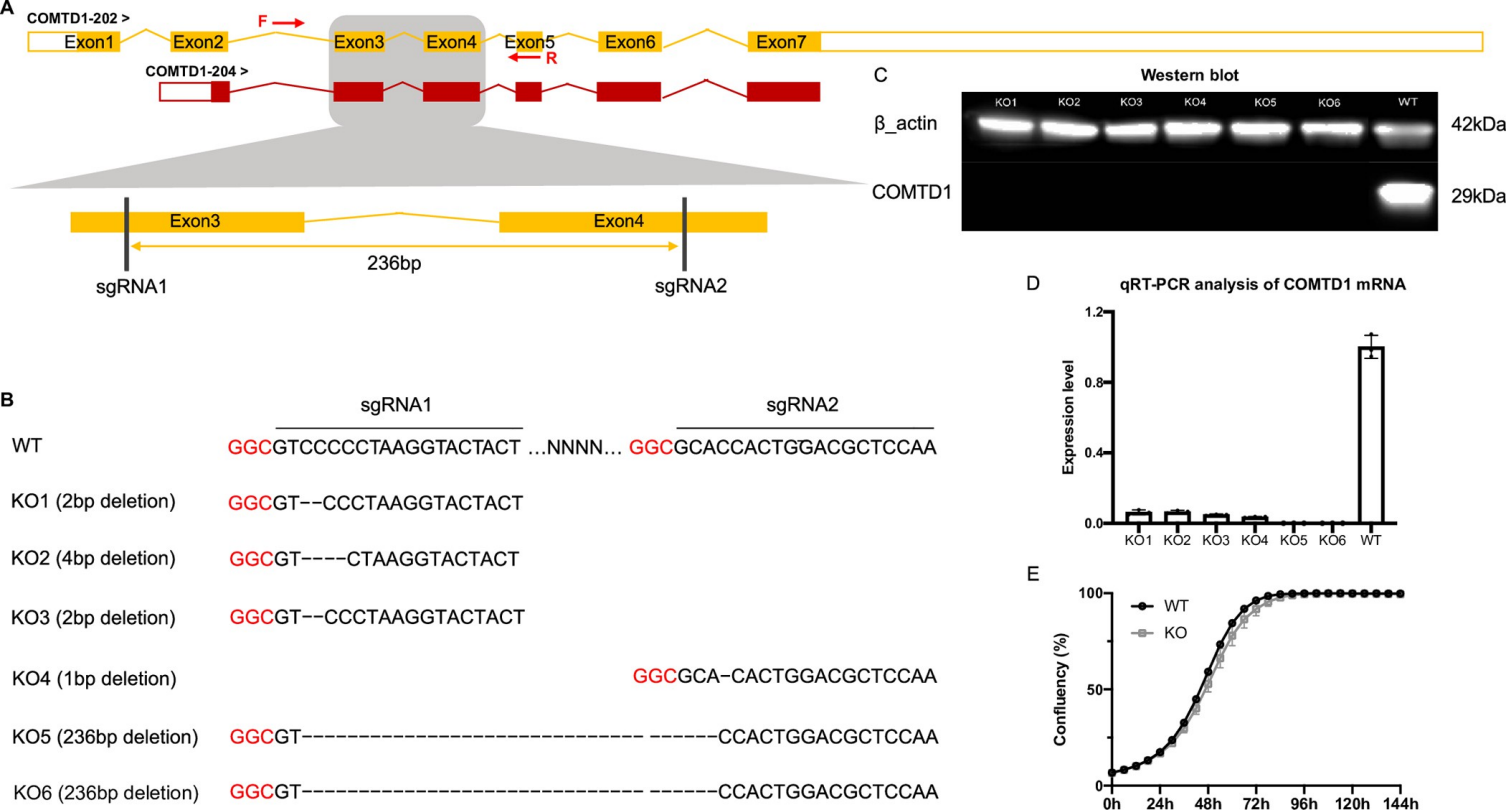
CRISPR/Cas9 mediated inactivation of *Comtd1* in B16F10 cells. (A) Schematic description of CRISPR/Cas9 mediated inactivation of murine *Comtd1*. Black bars indicate the target sites of two gRNAs in the exons of *Comtd1*. Yellow arrow indicates a 236bp deletion introduced by paired sgRNAs. The primer pair indicated by red arrows is used for amplifying genomic DNA. (B) Six colonies retrieved from *Comtd1* knockout in B16F10 cells. The PAM sequence is in red. Dash (-) indicates deleted nucleotides. The top one is wild type and the others are KO clones, of which, three lines (KO1, KO2, KO3) were generated using sgRNA1, one line was generated using sgRNA2, and the last two lines carrying 236bp deletion were generated by the sgRNA pair. (C) Western blot analysis of whole-cell lysates prepared from six *COMTD1* knockout clones and one WT cell line using antibodies against COMTD1 and the control β-actin. (D) Quantitative RT-PCR analysis of *Comtd1* expression in KO and WT cell lines. Date are presented as mean ± SD (n = 3 biological replicates). (E) Cell growth curves of KO (grey) and WT (black) were recorded by the Incucyte Zoom live-cell imaging system and data are expressed as cell confluence (%; mean ± SEM, n = 6 in KO, n = 3 in WT).

To assess the impact of *COMTD1* on cellular metabolism and explore links between metabolism and melanogenesis, we used CRISPR/Cas9-mediated gene targeting to generate a *Comtd1* knockout (KO) in the pigmented mouse B16F10 melanoma cell line (**Fig 6A**). We targeted exons 3 and 4 that are present in both chicken *COMTD1* transcripts and that are expected to encode part of the *O*-methyltransferase domain. Cells were transfected with Cas9 and either a single sgRNA or paired-gRNAs to generate different knockout clones. PCR screening of B16F10 clones revealed four clones carrying deletions around either the sgRNA1 or sgRNA2 targeting site from cells transfected with single sgRNAs and two clones carrying a 236 bp deletion between sgRNA1 and sgRNA2 from cells transfected with both sgRNAs. *Comtd1* inactivation in these clones was validated by Sanger sequencing, RT-qPCR analysis, and immunoblotting (**Fig 6B–6D**). Growth curves of WT and KO cell lines in standard culture medium over six days did not differ significantly (**Fig 6E**), suggesting that loss of *Comtd1* does not substantially impact cell viability, in accordance with the phenotype of *Comt-*deficient mice [51].

**Fig 7:**
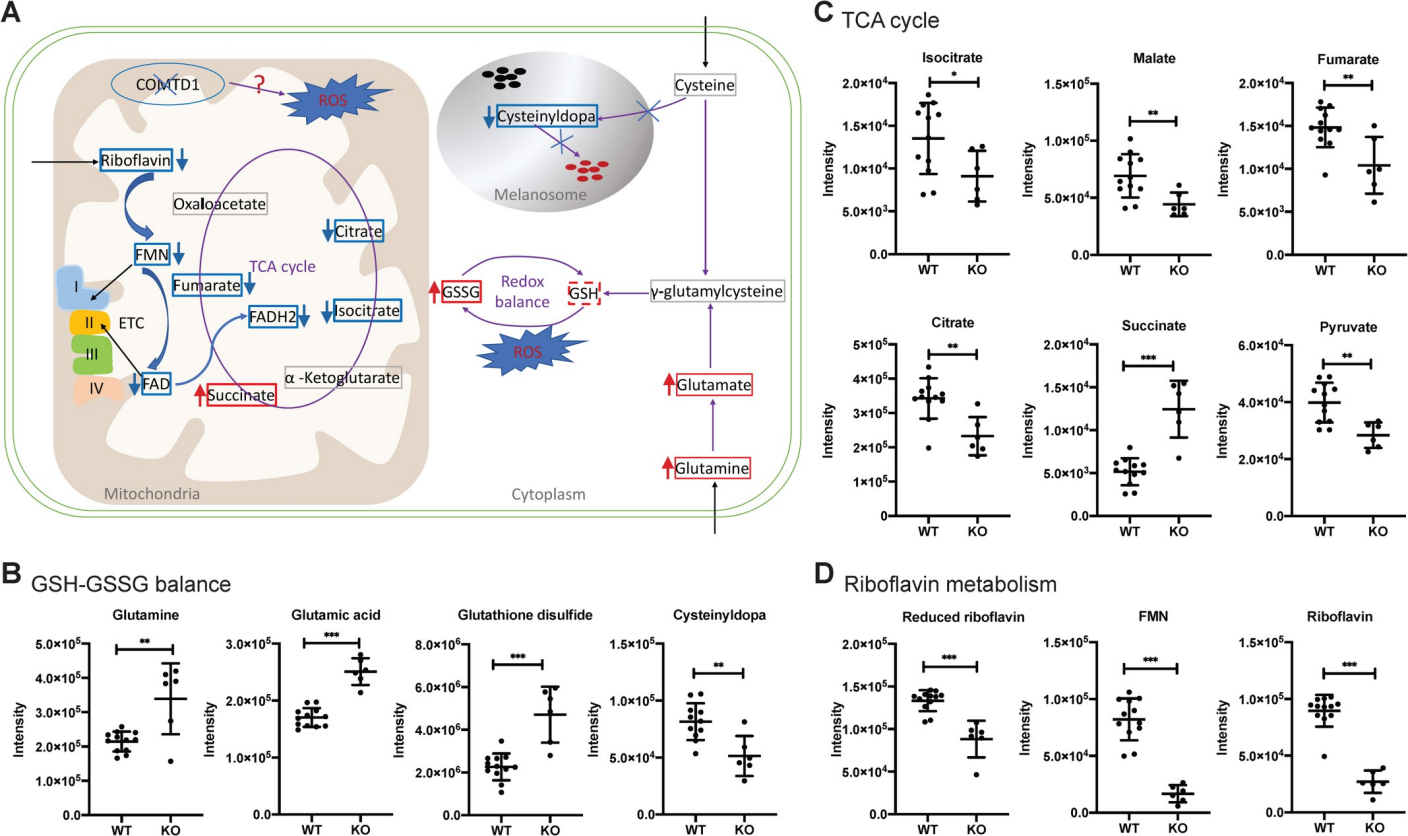
Metabolomics analysis reveal several metabolic pathways involved in oxidative stress that are altered in *Comtd1*-KO B16F10 cells. (A) Schematic representation of possible impact on biosynthesis of pheomelanin by COMTD1. Upregulated metabolites are highlighted in red and downregulated in blue. The solid and dashed lines denote significant and nonsignificant difference, respectively. (**B-D)** Mass spectrometric peak intensity of the corresponding metabolites in wild-type and knockout cell lines. Data are presented as mean ± SD from experimental replicates (N = 6 in KO; N = 12 in WT). ns: not significant, P > 0.05; *: P < 0.05; **: P <0.01; ***: P <0.001. Peak intensity of metabolites was acquired by UPLC-MS analysis.

To determine if COMTD1 impacts mitochondrial metabolism, we used ultra-high pressure liquid chromatography fractionation and mass spectrometry (UPLC-MS) to compare the metabolic signatures of WT and *Comtd1* KO B16F10 cells. Unsupervised multivariate analysis (PCA) was used to explore potential metabolic differences associated with *Comtd1* inactivation. Additionally, supervised multivariate analysis (PLS-DA) revealed a clear distinction in the metabolome of WT and *Comtd1* KO cells after cross validation (CV) of the model (**S3A–S3D Fig**). Metabolic pathway analysis documented significant differences in multiple metabolic pathways (**S3E Fig**), particularly those that impact responses to oxidative stress including glutamate/glutathione metabolism, riboflavin metabolism, and the tricarboxylic acid (TCA) cycle (**Fig 7**). Furthermore, we noted up-regulated levels of biochemical indicators of cell redox balance, glutathione (GSH) and glutathione disulfide (GSSG) (**Fig 7A**). The two upstream metabolites in glutathione biosynthesis, glutamine and glutamate, are also significantly up-regulated in the *Comtd1*-deleted cell lines (**Fig 7B and S5 Table**). The significant down-regulation of cysteinyldopa (**Fig 7B and S5 Table**), an intermediate in pheomelanin synthesis, is of particular interest in relation to the IG phenotype. The majority of metabolites involved in the Krebs cycle were significantly reduced in the *Comtd1* KO cells except succinate, which showed the opposite trend (**Fig 7C** and **S5 Table**), indicating impaired entry into the Krebs cycle and a block in succinate dehydrogenase—the only enzyme in the pathway that requires reduction of oxidized FAD to FADH2. This is consistent with the large reduction in riboflavin and FMN (**Fig 7D**), both precursors of FAD, and suggest a defect in mitochondrial function. In addition, the content of riboflavin, a key component of the mitochondrial respiratory chain (RC), was 3.4-fold reduced in *Comtd1* KO cells relative to wild-type cells (**Fig 7D**).

### *COMTD1* overexpression mitigates damage from cell stress in melanocytes *in vitro*

**Fig 8:**
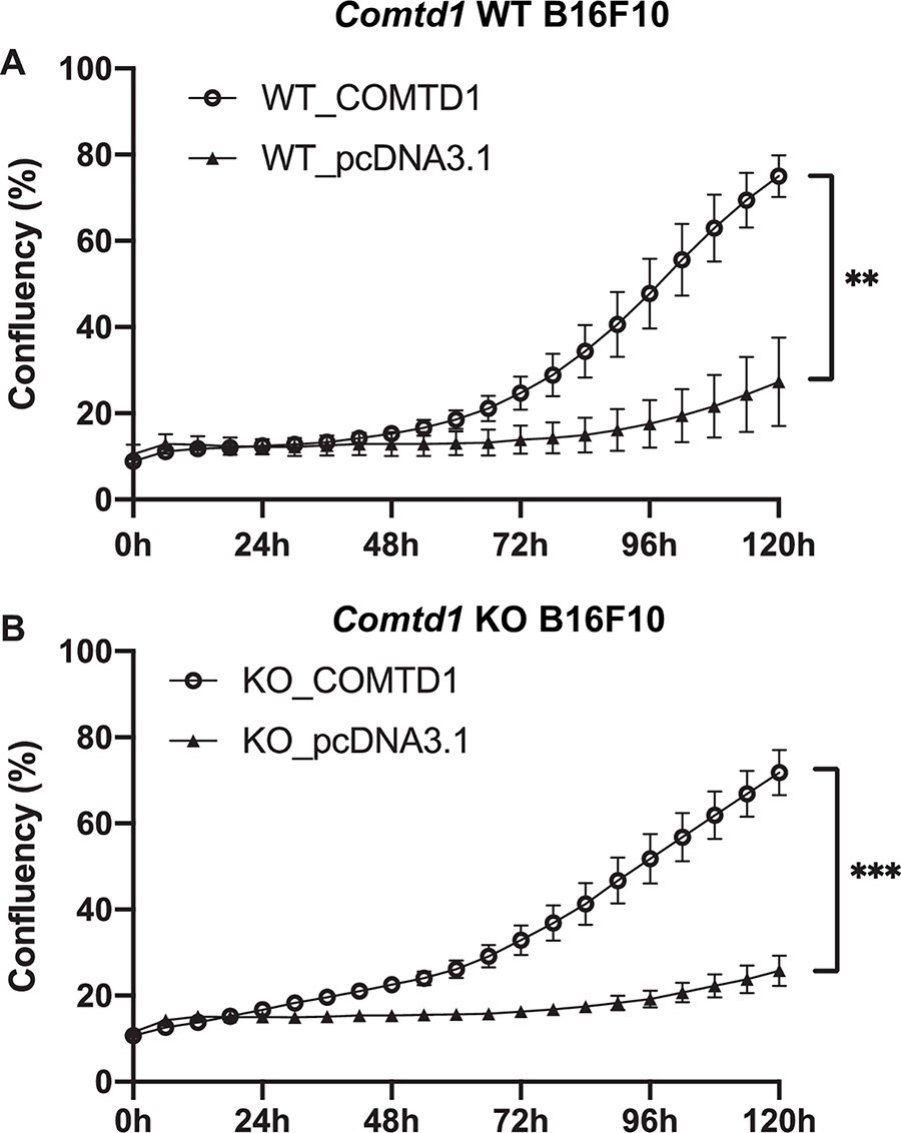
Proliferation curves of wild-type and *Comtd1* null B16F10 cell lines after transfection. A-B, Proliferation curves of *Comtd1* WT (A) and *Comtd1* KO B16F10 cell lines (B) after introducing either *COMTD1* expression vector (Circle) or its corresponding empty vector pcDNA3.1 (triangle). Growth was recorded by the Incucyte Zoom live-cell imaging system and data are expressed as cell confluence (%; mean ± SEM, n = 4 in KO, n = 2 in WT). **: P <0.01; ***: P <0.001.

Given the alterations in glutathione metabolites observed in *Comtd1* KO B16F10 cells and the critical role of glutathione in resistance to oxidative stress, we tested whether COMTD1 might impact cellular responses to stress. Chemical-mediated transfection methods have been reported to induce acute inadvertent toxic effects including oxidative stress [52–55]. We therefore treated wild-type and *Comtd1* KO melanocytes with jetPRIME transfection reagent and either empty pcDNA3.1 vector or pcDNA3.1 driving COMTD1 expression, and assessed growth over six days. Transfection of either WT or *Comtd1* KO cells with empty vector resulted in a drastically reduced growth rate compared with untreated cells (compare **Fig 6E** with **Fig 8**, filled triangles). By contrast, transfection with the COMTD1 expression vector strikingly restored wild-type growth rates to both WT and KO cells (**Fig 8A** and **8B**, respectively, empty circles). This indicates that overexpression of COMTD1 mitigates cellular stress caused by chemical transfection.

## Discussion

The dilution of red pigmentation in chickens homozygous for the *IG* mutation has been documented for over 90 years, but the responsible mutation had not been identified. This study provides evidence that the causal mutation for the IG phenotype is a 2-bp insertion in exon 5 of *COMTD1*, encoding a mitochondrial transmembrane protein with *O*-methyltransferase activity. The mutation results in a frame shift in the middle of the enzymatic domain, likely inactivating it. As a consequence, mitochondrial metabolism is altered, with a consequent increase in glutathione synthesis and potentially increased susceptibility to oxidative stress. The changes in mitochondrial metabolism affect pheomelanin production in melanosomes, likely indirectly. Together, the data suggest an important role for *COMTD1* in controlling the production of oxidative intermediates in melanocytes and thus for controlling melanin production.

Our genetic data showed that the *IG* locus mapped to a 433 kb interval on chicken chromosome 6 containing three protein-coding genes (*LRMDA*, *ZNF503*, and *COMTD1*) and two lncRNA genes (**Fig 3**); a single recombinant chromosome further reduced the IBD interval to 262 kb containing only *COMTD1*. The genetic dissection of the *IG* locus was hampered by an unusual low recombination rate in this chromosomal region resulting in poor resolution in linkage mapping and a relatively large IBD region. However, both *LMRDA* and *COMTD1* were candidates for a pigmentation phenotype. A nonsense mutation in *LMRDA* causes oculocutaneous albinism type 7 in humans [56], and knockout of *LRMDA* in a human melanoma line causes alterations in PMEL processing and in the expression of melanogenic genes [57], suggesting that *LRMDA* plays an important role in eumelanin synthesis. However, disruption of this gene was not expected to preferentially impair pheomelanin production. *COMTD1* was considered an *IG* candidate because the protein encoded by its paralog, *COMT*, has been shown *in vitro* to catalyze *O*-methylation of the melanin substrate L-Dopa to 3-*O*-methoxytyrosine [58]. Due to the large IBD interval we restricted our search for a candidate causal mutation to the three protein-coding genes, and identified a 2-bp insertion in exon 5 of *COMTD1*. While we cannot formally exclude that non-coding changes in the interval cause or contribute to the phenotypic effect, causality of the 2-bp insertion is supported by (i) a complete genotype-phenotype correlation across chicken breeds in which no chicken with a clear non-IG phenotype was homozygous for the 2-bp insertion, and (ii) the dramatic reduction in pheomelanin metabolites detected in mouse B16F10 cells with a targeted inactivation of *Comtd1* (**Figs 7 and S3**). The nature of this mutation and the functional data presented in this study make the inactivation of *COMTD1* a plausible causal mutation for the IG phenotype in the chicken. This is the first *COMTD1* mutation associated with a phenotypic effect in any organism.

Two different COMTD1 transcripts were detected in IG birds: a full-length transcript (IG1), which is out of frame after the 2 bp insertion in exon 5 leading to a premature stop codon, and an alternative splice form (IG2), which carries four out-of-frame codons from exon 5, lacks exon 6, but contains exon 7 in frame. It is likely that this splice variant is only noted in IG birds because the full-length transcript is degraded by nonsense-mediated RNA decay [59] allowing a rare splice variant to be visible after standard PCR.

*COMTD1* orthologs are present in all vertebrates. However, while the *O*-methyltransferase domain in COMTD1 shows high sequence identity to the related COMT, a molecular function for the COMTD1 protein has not yet been established. Our findings that COMTD1 localizes to mitochondria in immortalized melanocytes (**Fig 5**) and that inactivation of *Comtd1* in a mouse melanoma cell line alters the pattern of mitochondrial metabolites (**Fig 7**) provides the first evidence that COMTD1 regulates mitochondrial metabolism. Thus, our work adds to the growing list of links between mitochondrial function and melanin production in melanocytes [60–64].

Many genes are known to regulate eumelanin synthesis or the switch between eumelanin and pheomelanin [65], but only a few have been identified that specifically affect pheomelanin synthesis. Mutations in *Slc7a11*, *Ggt1*, *Mfsd12*, *Clcn7*, *Ostm1*, *Sox10*, and several as yet undefined genes have all been shown to reduce pheomelanin pigmentation in mouse with no or modest impact on eumelanin production [10,66–72]. Several of these genes (*Slc7a11*, *Mfsd12*, and *Ggt1*) impact cysteine uptake, intracellular transport, or incorporation into glutathione [10,73,74], and thus might share a mechanistic basis with *Comtd1* for their impact on pheomelanin synthesis. *Clcn7* and *Ostm1* encode the subunits of the CLC7 chloride transporter that impacts lysosomal pH [75], and thus might alter the pH of melanosomes promoting specifically pheomelanin synthesis. In chicken, two different missense mutations in the *SLC45A2* gene are associated with a specific inhibition of pheomelanin pigmentation characteristic for the sex-linked Silver phenotype [49], while *SLC45A2* mutations in other organisms inhibit both eumelanin and pheomelanin pigmentation [76–78]. Like CLC7, SLC45A2 regulates melanosome pH [79], but how these *SLC45A2* mutations in chicken cause this specific effect on pheomelanin production is unclear.

COMTD1 is an *O*-methyltransferase based on the presence of the conserved *O*-methyltransferase domain, the binding of *S*-adenosylmethionine in the crystal-structure (pdb: 2AVD, https://www.rcsb.org/structure/2AVD), and its structural and sequence similarity to COMT. *COMT* has been implicated the protection of melanocytes from the cytotoxic dihydroxyindole derivatives that are formed during eumelanogenesis by *O*-methylation [31]; indeed, *O*-methylated forms of the eumelanin intermediates 5,6-dihydroxyindole and 5,6-dihydroxyindole-2-carboxylic acid have been detected in the urine and serum from human melanoma patients and melanoma-bearing mice [44,80–83]. The pheomelanin precursor 5-*S*-cysteinyldopa is a known target of *O*-methylation [84] and its presence in urine is a diagnostic marker for human melanoma [85]. *COMTD1* may have higher specificity to toxic compounds that are formed during pheomelanin synthesis, and thus may protect pheomelanogenic melanocytes from damage or death; indeed, such an effect might have been missed in our experiments using B16F10 cells, which predominantly generate eumelanin [86]. If this is the case, then one might predict that pheomelanogenic IG birds will have fewer functional melanocytes than wild-type birds.

Inactivation of *Comtd1* in mouse melanocytes had a significant effect on cysteine and methionine metabolism (**S3 Fig**). Thus, mutations in *COMTD1* may affect the synthesis of pheomelanin indirectly by regulating the levels of cysteine through the conversion of its cofactor *S*-adenosylmethionine (SAM) to *S*-adenosylhomocysteine (SAH). SAH can in turn either be metabolized to homocysteine and subsequently to cysteine or be converted to methionine. Polymorphisms in human *COMT* are associated with decreased levels of homocysteine in blood plasma, and extensive usage of COMT inhibitors is associated with low plasma homocysteine levels [87,88]–which positively correlate with cysteine levels in human [89]. It is possible that a non-functional COMTD1 might result in decreased levels of homocysteine and cysteine in the plasma and thereby inhibit pheomelanogenesis due to limited cysteine accessibility in melanocytes. Such a mechanism would be expected to have a more broad impact on cellular physiology, which is not observed in IG birds [90], but may be compensated by other metabolic alterations in specific cell types. By contrast, melanocytes are highly vulnerable to oxidative stress, and thus might be more sensitive to decreased cysteine concentration than other tissues. During periods of oxidative stress, the limited supply of cysteine in melanocytes may be preferably used to generate glutathione and maintain a healthy redox balance at the cost of reduced pheomelanin synthesis; this hypothesis is supported by the elevated GSH level and decreased cysteinyldopa level observed after *Comtd1* inactivation in B16F10 cells (**Fig 7B and S5 Table**).

Further functional studies of the *IG* mutation are needed to fully understand how the mutation affects the synthesis of pheomelanin. The IG bird may also be used as a model to investigate the importance of *COMTD1* in other physiological processes. We noted a 20% reduction of the number of *IG/IG* homozygotes at birth in the F2 generation used for linkage mapping, 20% compared with the expected 25%. Given that we have documented that the *COMTD1 CT* insertion shows complete concordance with the IG phenotype and serves as a diagnostic marker for it, we predict that the reduction in live *IG/IG* homozygotes is not due to incomplete penetrance but rather to mild sub-lethality due to an important role for *COMTD1* outside the pigment system.

## Materials and methods

### Ethics statement

The animal experiments was authorised under number 02410–02 by the French Ministry for Agriculture after advice from the INRA Val de Loire ethical committee for animal experimentation.

### Animals

DNA samples from a pedigree developed at the PEAT Poultry Experimental Facility (INRAE, Nouzilly; https://doi.org/10.15454/1.5572326250887292E12) were used for the linkage analysis. The pedigree comprised a three-generation intercross between homozygous carriers of the *IG* allele and RIR birds, non-carriers of this allele, which included 159 informative meiosis for the *IG* locus. This intercross was part of a larger programme to maintain a resource flock for the analysis of morphological variation.; The homozygous *IG/IG* birds, representing five different populations, used in the Identical-By-Descent (IBD)–mapping were collected by Bertrand Bed’hom and Michèle Tixier-Boichard except for the SASSO IG-line that was provided by SASSO (a French breeding company). The genotype data of the wild-type chickens at the *IG* locus used in the IBD-mapping, were all downloaded from published data [91]. The genome-sequence of the *IG/IG* bird was generated from a chicken from the same INRA population that was used to set up the mapping pedigree. Most of the wild-type birds used in the genotyping of the 2-bp insertion were collected under the Avian Diversity (AvianDiv) European project [92]. The Smyth Line, Brown Line samples and samples of the Obese-strain/ red junglefowl pedigree material were from birds kept in Uppsala, Sweden. The Ceylon and Grey junglefowl samples were provided by Clères Zoological Park, France, and Dr A. Fumihito and Dr. M. Nishibori, Japan, respectively.

Feathers from R+ and R- lines were included to illustrate phenotypic variation in pigment intensity among birds exhibiting a red plumage without carrying the *IG* allele. These lines have been selected since 1975 and exhibit since the 1990s a difference in feather colour: the least efficient line, R+, exhibits a darker plumage than the more efficient one, R-. This was tentatively related to a higher heat production in R+ birds than in R- birds, on the basis that a darker colour increases heat exchanges with the environment and thus facilitates heat dissipation. The two lines were also shown to differ in mitochondrial activity in semen, with a lower activity in the R+ line, due to a lower number of mitochondria [93].

### Chemical characterization of melanin

Feather samples (20–25 mg) were cut from the neck of Rhode Island Red (R+ and R-), and of IG birds. Cutting did not create pain as would pulling of feathers do, because only the non-growing part of the feather was cut. Therefore, this procedure did not require an ethical permit. Samples were homogenized in water with Ten-Broeck glass homogenizer at a concentration of 10 mg/mL. Aliquots (100 μL) were subjected to Soluene-350 solubilization [45], alkaline hydrogen peroxide oxidation (AHPO) [46] and HI hydrolysis [47]. Soluene-350 solubilization affords A500 and A650 values. Absorbance of 0.021 and 0.001 due to proteins per mg feathers were subtracted from A500 and A650 values. As a pheomelanin prepared from dopa and cysteine gave A500 value of 4.26 per mg, we calculate a conversion factor for pheomelanin of 235 (1000/4.26). Eumelanin, benzothiazine-pheomelanin, and benzothiazole-pheomelanin contents were calculated by multiplying those of PTCA, 4-AHP, and TTCA by factors of 38, 7 [94], and 34 [95], respectively.

### Linkage analysis

Linkage analysis was performed using the CRIMAP software version 2.4 [96]. Pyrosequencing was used to assay four SNPs and three microsatellite markers were analysed using the MegaBACE capillary electrophoresis instrument (GE Healthcare, Uppsala). PCR and sequencing primer sequences together with positions of the assayed genetic makers are listed in **S6 Table**. Genotypes at the 2-bp insertion locus were determined using a custom TaqMan SNP Genotyping assay (Applied Biosystems, CA, U.S.A).

### Identical-by-descent mapping

Partial re-sequencing was performed on homozygous IG birds (*IG/IG*) and wild-type birds (*N/N*), and included chromosomal regions spread across the interval showing no recombination within the *IG* locus. Additional sequencing of the same panel of birds in regions located in close proximity to *COMTD1* was later performed to reduce the IBD-region. All primers were designed using the PRIMER3 plus software [97] and DNA sequences were analyzed and edited with CODONCODE ALIGNER 3.7.1. (CodonCode, Dedham, MA, USA). Primer sequences are listed in **S6 Table**.

### Whole genome resequencing

DNA from an IG bird was prepared for sequencing. Illumina paired-end libraries were generated from these DNA samples (mean insert sizes of approximately 220 bases). The library was sequenced on two lanes using an Illumina HiSeq instrument (Illumina, San Diego, U.S.A.) according to the manufacturer’s instructions. The reads were mapped to the chicken genome (galGal6 genome assembly) using the software BWA (version: 0.7.12) [98] resulting in average read depths of approximately 30X over the chicken genome. Following removal of duplicates using the Picard toolkit [99] SNPs and small insertions/deletions were identified from the alignment files using SAMtools (version 1.6) [100] in combination with custom python scripts. The mapping data were used to determine read depths in 1 kb windows over the region of interest. The mapping distances between mate-pairs were used to detect structural variation in relation to the reference assembly.

### Analysis of publicly available whole genome sequence (WGS) data

Publicly available WGS data from 172 individuals or pooled samples were analyzed (**S4 Table**). It included 45 samples from chickens expressing red plumage color, and 127 samples from chickens with known plumage color. All these Illumina paired-end FASTQ data were aligned to the galGal6 genome assembly using BWA (version: 0.7.12) [98] sorted with SAMtools (version: 1.6) [100] and candidate variants were called with GATK HaplotypeCaller 3.8 [101].

### Intersection of polymorphism with functional sequences

The intersection of polymorphisms with evolutionary conserved and exon sequences were performed using the Galaxy software [102]. The nucleotide positions of the exons were based on ENSEMBL predictions.

### Alignment of protein sequences

The sequences of *COMTD1* orthologs were downloaded from the NCBI protein database (Chicken, XP_040530512; Zebra Finch, XP_002192673; Anolis lizard (*Anolis carolinensis*), XP_003223173; pufferfish, CAG04823; dog, XP_546175; human, NP_653190; salmon, ACI67838; mouse, NP_081241). The first 87 amino acids in the chicken predicted COMTD1 protein sequence (XP_040530512) were not included in the alignment because this sequence did not show sequence identity to COMTD1 sequences from other species. The alignment was performed using Clustal Omega [103].

### Plasmid construction and antibodies

Mouse *Comtd1* (NCBI reference sequence NP_081241) was synthesized (GenScript Inc, Piscataway, NJ, USA) with a hemagglutinin HA11-epitope tag sequence fused in-frame to either the 5’ (HA-COMTD1) or 3’ (COMTD1-HA) end of the *COMTD1* coding sequence, and subsequently cloned into the vector pcDNA3.1(-) (Invitrogen) using the *Nhe*I and *Not*I restriction sites.

Monoclonal antibodies and their sources include: mouse anti-TYRP1 (TA99, a.k.a. Mel-5) from American Type Culture Collection; mouse anti-PMEL clone HMB45 from Enzo; mouse anti-MAVS clone C-1 (sc-365333) from Santa Cruz; and rat anti-HA11 clone 3F10 from Sigma. Polyclonal antibodies generated in rabbits included: rabbit anti-STX13 [104], as previously described [105]; AbCam antibodies to LAMP2 (# ab18528), calnexin (#ab22595), beta actin (#ab8227), and COMTD1 (#ab228014). Highly absorbed, species-specific secondary antibodies from donkey and conjugated to Alexa Fluor 488 and 594 were from Jackson ImmunoResearch Laboratories, Inc; the secondary donkey anti-rabbit HRP-conjugated antibody (NA9340V) was from GE Healthcare.

### Cell culture

Immortalized melan-Ink4a melanocytes derived from C57BL/6J-Arf-*Ink4a*^−/−^ (*Cdkn2a* null) mice [106] were cultured in RPMI 1640 medium (Invitrogen or Corning) supplemented with 200 nM 12-O-tetradecanoylphorbol-13-acetate and 10% FBS (Atlanta Biologicals) in a humidified atmosphere with 10% CO_2_. The B16F10 mouse melanoma cell line was cultured in DMEM supplemented with 10% FBS and 100 U/ml penicillin, 100 μg/ml streptomycin (GIBCO) at 37°C with 5% CO_2_.

### Immunofluorescence microscopy

Melan-Ink4a melanocytes were seeded at 7 x 10^5^ in 35 mm wells on Matrigel (Corning) coated coverslips and transfected 24–36 h later with 8 μL Lipofectamine 3000, 1 μg of carrier DNA (pCI plasmid, Clontech), and 1 μg of HA-COMTD1 or COMTD1-HA plasmid. Cells were analyzed by immunofluorescence microscopy 18–24 h later. Immunofluorescence labelling was performed as described [107]. Briefly, melan-Ink4a melanocytes on coverslips were fixed in PBS/ 2% formaldehyde for 20 min, blocked and permeabilized in blocking buffer (PBS, 0.1% BSA, 0.02% saponin), and then labelled with primary antibodies diluted in blocking buffer for 1 h at RT. Cells were washed 3 x 5 min with PBS, and then incubated with secondary antibodies diluted in blocking buffer for 30 min at RT. Samples were washed for 15 min in PBS, mounted with Prolong Gold Antifade Mountant (Invitrogen), and analyzed as described [108] by epifluorescence microscopy on a DMI 6000B microscope (Leica Biosystems) equipped with a 63X Plan Apochromat objective (1.4 NA) and a Hamamatsu Photonics ORCA-Flash 4.0 sCMOS digital camera. Both fluorescence and bright field images were captured. Images were acquired as a z-series with 0.19 μm steps, and were deconvolved using the blind deconvolution algorithm of Microvolution software (BioVision Technologies) and ImageJ [109].

### Quantitative microscopy analysis

The area of overlap between two fluorescently labelled proteins or between fluorescently labelled proteins and pigmented melanosomes was quantified using ImageJ [109] on deconvolved fluorescence images essentially as described [108]. Briefly, single cells were cropped and the fluorescence in the perinuclear region was removed. Prior to thresholding, local background was subtracted using a rolling ball radius of 2 pixels and a sliding paraboloid. Binary images were created by subtracting background using the subtract operation before applying the Bernson auto local threshold algorithm in ImageJ. The ImageJ Image Calculator plugin was used to multiply two binary images together to create an image representing the area of overlap between the two fluorescent markers or between one fluorescent marker and melanin in the bright field channel. Structures in the original and overlap binary images larger than 0.2 μm^2^ were counted using the Analyze Particles plugin, and the ratio of overlap pixels to total fluorescent pixels of the imaging channel of interest was used to generate the percent overlap between two channels. Results represent values obtained from at least ten cells from at least three individual experiments.

### Generation of *Comtd1* knock-out cell lines

CRISPR/Cas9 induced mutagenesis was done as previously described [110]. Briefly, sgRNAs targeting functional domain of *COMTD1* (sgRNA1: GTCATCATGGAATCCCCCTG, sgRNA2: AACCTCGCAGGTCACCACG) were computationally identified (http://www.genome-engineering.org/crispr) and inserted into the pSpCas9 (BB)-2A-GFP (PX458) vector (Addgene plasmid #48138). B16F10 cells were seeded in a 6-well plate 12 h before transfection. Two μg of plasmid and 8 μl jetPRIME reagent (Polyplus transfection) were transfected into melanocytes according to the manufacturer’s protocol. B16F10 cells were harvested at 48 h post-transfection and single GFP-positive cells were sorted in a cooled 96 well plate. Around two weeks later, single clones were collected and the sequence of target region within *Comtd1* were amplified and examined by Sanger sequencing. Cell lines carrying in-frame shift mutations were further verified by quantitative PCR and western blot analysis. Primer sequences are listed in **S6 Table**

### RNA extraction, RT-PCR and quantitative RT-PCR

Total RNA was extracted from feather follicles representing the three genotypes at the *IG* locus using the RNAeasy Mini KIT (Qiagen). cDNAs were synthesized using The High Capacity cDNA Reverse Transcription Kit (Applied Biosystems) and then used as template to amplify *COMTD1* transcripts. *COMTD1* transcripts were examined by gel electrophoresis and verified with Sanger sequencing.

Total RNA isolated from B16F10 cells were reverse-transcribed into cDNA and analyzed by qPCR on ABI7500 Fast Real-Time PCR System (Thermo Scientific) using SYBR PCR master mix (Thermo Scientific). Ct value was first normalized to the housekeeping gene *Hprt*, then the average expression of *Comtd1* in the WT cell line was assumed to be 1 for the subsequent calculation of the relative expression in KO cell lines. Primer sequences are available in **S6 Table.**

### Protein extraction and Western Blot analysis

WT and KO B16F10 cells were washed by cold PBS twice and then harvested in RIPA buffer (Thermo Scientific) containing Protease and Phosphatase Inhibitor (Thermo Scientific). Cell extracts were centrifuged at 13,000g at 4°C for 15 min, and the supernatants were collected. Protein concentrations were quantified by the BCA protein assay kit (Thermo Scientific). Twenty ng protein from each cell line was aliquoted and heated at 95°C for 5 min after mixing with Laemmli Sample Buffer (Bio-Rad). Proteins were resolved by Tris-Glycine SDS-PAGE and were transferred to PVDF membranes (Bio-Rad). All membranes were incubated with specific antibodies overnight at 4°C. Primary antibodies were detected with horseradish peroxidase-conjugated secondary antibodies followed by exposure to ECL reagents (Thermo Fisher Scientific) and visualized by ChemiDoc MP Imaging systems (Bio-Rad).

### Metabolomics

Solvents and reagents were purchased from Sigma-Aldrich or Fisher Scientific and were used without further purification. Authentic standards were also purchased from Sigma-Aldrich or Fisher Scientific. The in-house built metabolite library was obtained from MetaSci. Mass spectrometry grade solvents were used for UPLC-ESI-MS analysis.

Each sample containing 1 x 10^6^ cells was seeded in 100 mm plates 36 h before harvest. Cells were washed three times with ice cold PBS and the residual buffer was removed by vacuum until dryness. The samples were quickly quenched by adding HPLC-grade MeOH in the plates, and collected by detaching quenched cells with a rubber-tipped cell scraper. The mixtures were aliquoted and stored at -80°C until metabolites extraction.

Aliquoted samples (25 μL) were transferred into microplate well for protein quantification using Pierce BCA Protein Assay Kit strictly following the user guide. The UV absorbances were measured at 562 nm on a plate reader (VarioskanFlash).

After the aliquoted cell cultures were taken from -80°C freezer, they were placed on ice for 2 h for thermal equilibration. MilliQ-H_2_O (356 μL) was added to Eppendorf tubes containing cell samples (500 μL) followed by adding HPLC-grade chloroform (500 μL). The mixture was incubated at ThermoMixer (4°C, 20 min, 1,400 rpm) before the centrifugation (4°C, 5 min, 16,100 g). The aqueous phases were transferred into new Eppendorf tubes and the solvent was removed under reduced pressure in a vacuum concentrator (Speedvac). The residue was redissolved in 5% *v/v* acetonitrile/ H_2_O solution according to protein quantification results (**S7 Table**) for metabolite normalization before UPLC-MS analysis submission.

The UPLC-MS/MS analysis was performed in a SYNAPT G2-S High-Definition Mass Spectrometer (HDMS) using an electrospray ionization (ESI) source with an AQCUITY UPLC I-class system and equipped with a Waters ACQUITY UPLC HSS T3 column (1.8 μm, 100 × 2.1 mm). Water with 0.1% formic acid was used as mobile phase A and methanol with 0.1% formic acid was used as mobile phase B. The column temperature was kept at 40°C, and the autosampler at 6°C. The flow rate was set to 0.2 mL/min. The gradient used was as follows: 0–8.5 min, 0–100% B; 8.5–10 min, 100% B; 10–11 min, 100–0% B; 10–15 min, 0% B. The system was controlled using the MassLynx software package v 4.1 from Waters. High-resolution mass spectra were acquired in positive and negative ionization mode, at a mass range of *m/z* (mass-to-charge ratio) 50–1,500. Data acquisition was performed in MSE mode. The samples were injected to the UPLC-MS system in a randomized order with QC samples.

Significant features and molecules of interest were primarily annotated by databases (www.hmdb.ca, https://metlin.scripps.edu/) based on their *m/z* value and given the high mass accuracy provided by the mass analyzer. Subsequently, in-house built standard library or purchased standards, measured in the same UPLC-MS/MS system, were used for the assignment of the retention time (rt).

The chromatograms and mass spectra were processed using the XCMS R package for peak alignment and retention time correction in both positive and negative ionization mode. An overview of the data was provided by principal component analysis (PCA), prior to which the data was auto-scaled using the metabolomics platform (www.metaboanalyst.ca). Partial Least-Squares Discriminant Analysis (PLS-DA) is a supervised multivariate analysis with the full awareness of the class labels. This method can be used for feature selection after cross-validation (CV). The normality of the test statistics and *P*-values were evaluated using the same platform and the data were distributed normally. For the hypothesis testing, two-tailed t-test was applied in metabolites extracted from *Comtd1* knockout and wild-type cell cultures for detecting metabolic differences. Pathway analysis were performed in the same platform by inputting the compound names of significantly altered metabolite.

### Cell proliferation rate assay

For the cell proliferation rate assay, wild-type and six *Comtd1* knockout B16F10 cell lines were seeded in 96-well plates at a density of 5,000 cells/well in 100 μL growth media (with the supplement of 10% FBS). After overnight incubation, cells were treated in different ways. To measure the proliferation rate of WT and KO cell lines, cells were changed to the fresh media and the plates were placed in Incucyte Zoom live-cell imaging system for 6 days and the cell density were measured every 6 h. To validate the effect of overexpression of *COMTD1*, cells in each well were transfected with either 100 ng *COMTD1* expression vector (GenScript:OMu03356) or empty vector pcDNA3.1+/C-(K)-DYK (GenScript) as a negative control using 0.2 μl jetPRIME transfection reagent (Polyplus). Cells were cultured in Incucyte Zoom live-cell imaging system for 5 days and the cell density was measured every 6 h. The values obtained from at least three individual experiments were analyzed and displayed using GraphPad 8.

## Supporting information

S1 Fig Chemical characterization of hair melanin. **(**A) Levels of total melanin in wild-type birds (R+ and R-) and IG birds analyzed by Soluene-350 solubilization. (B) A650/A500 ratios analyzed by Soluene-350 solubilization. (C), eumelanin (EM), benzothiazine-pheomelanin (BT-PM), and benzothiazole-pheomelanin (BZ-PM) analyzed as PTCA, 4-AHP, and TTCA, respectively. Feather samples were obtained from neck regions from 3 males and 3 females. Results are shown with the means ± SEM of 3 birds. ns: not significant, P > 0.05; *: P < 0.05; **: P <0.01; ***: P <0.001 (Student’s t test).
(PDF)

S2 Fig HA-COMTD1 does not localize to melanosomes or endolysosomes. Immortalized melan-Ink4a cells were transiently transfected to express COMTD1 fused with the HA11 epitope at the *N*-terminus (HA-COMTD1) (A-D) or the HA11 epitope at the *C*-terminus (COMTD1-HA) (E-H). Two days later, cells were fixed and analyzed by bright field (BF) and immunofluorescence microscopy for HA and markers of either mature melanosomes (TYRP1; A, E), early stage melanosomes (PMEL; B, F), late endosomes/ lysosomes (LAMP2 C, G), or early endosomes (STX13; D, H). Individual images of labeled cells or the bright field image are shown in addition to an overlay of HA (green) with the indicated marker (red). Insets show a 7-fold magnified image of the boxed region to emphasize the lack of overlap. Main scale bar, 10 μm; inset scale bar, 2 μm.
(PDF)

S3 Fig Metabolomics analysis of *Comtd1*-KO and wild-type B16F10 cells. Principal component analysis (PCA) and Partial Least-Squares Discriminant Analysis (PLS-DA) with model validation results. PCA is an unsupervised multivariate analysis, which provides an unbiased overview of the metabolite features due to unawareness of the two groups compared (WT and KO in this case). PLS-DA is a multivariate analysis which considers data from the two groups and select the most discriminating metabolites that separates the two groups. This is the reason why PLS-DA show a better separation between WT and KO groups. (A) PCA in MS positive mode detection. (B) PLS-DA in MS positive mode detection. (C) PCA in MS negative mode detection. (D) PLS-DA in MS negative mode detection. (E) Significantly altered pathways impacted by *Comtd1* knockout.
(PDF)

S1 Table Sequence variants (non-reference alleles) in the coding region of *LRMDA*, *ZNF503*, and *COMTD1* detected by sequencing one homozygous *IG/IG* bird and the corresponding allele frequencies among non-IG *(N/-*) birds. (DOCX)

S2 Table The pheomelanic phenotype distribution in a White Leghorn (OS-line) X red junglefowl pedigree material according to genotype of the 2-bp-insertion (*TC*) in *COMTD1*. (DOCX)

S3 Table Genotype distribution of the 2-bp-insertion in *COMTD1* associated with the Inhibitor of Gold (IG) phenotype in different populations sorted by phenotype. Results deduced from whole genome sequencing data from the individuals listed in S4 Table.
(DOCX)

S4 Table Public whole genome sequence data from chicken used in this study. (DOCX)

S5 Table Detailed data for metabolic changes in the TCA cycle, the GSSG-GSH balance, and cysteine and methionine metabolism. (DOCX)

S6 Table Primer sequences used in this paper. (DOCX)

S7 Table Protein quantification results with experimental triplicate. (DOCX)
