# Supplementary figures and images for: A frame-shift mutation in COMTD1 is associated with impaired pheomelanin pigmentation in chicken
Source: PLoS Genet. 2023 Apr 17;19(4):e1010724. doi: 10.1371/journal.pgen.1010724 (PMC10138217; doi:10.1371/journal.pgen.1010724)

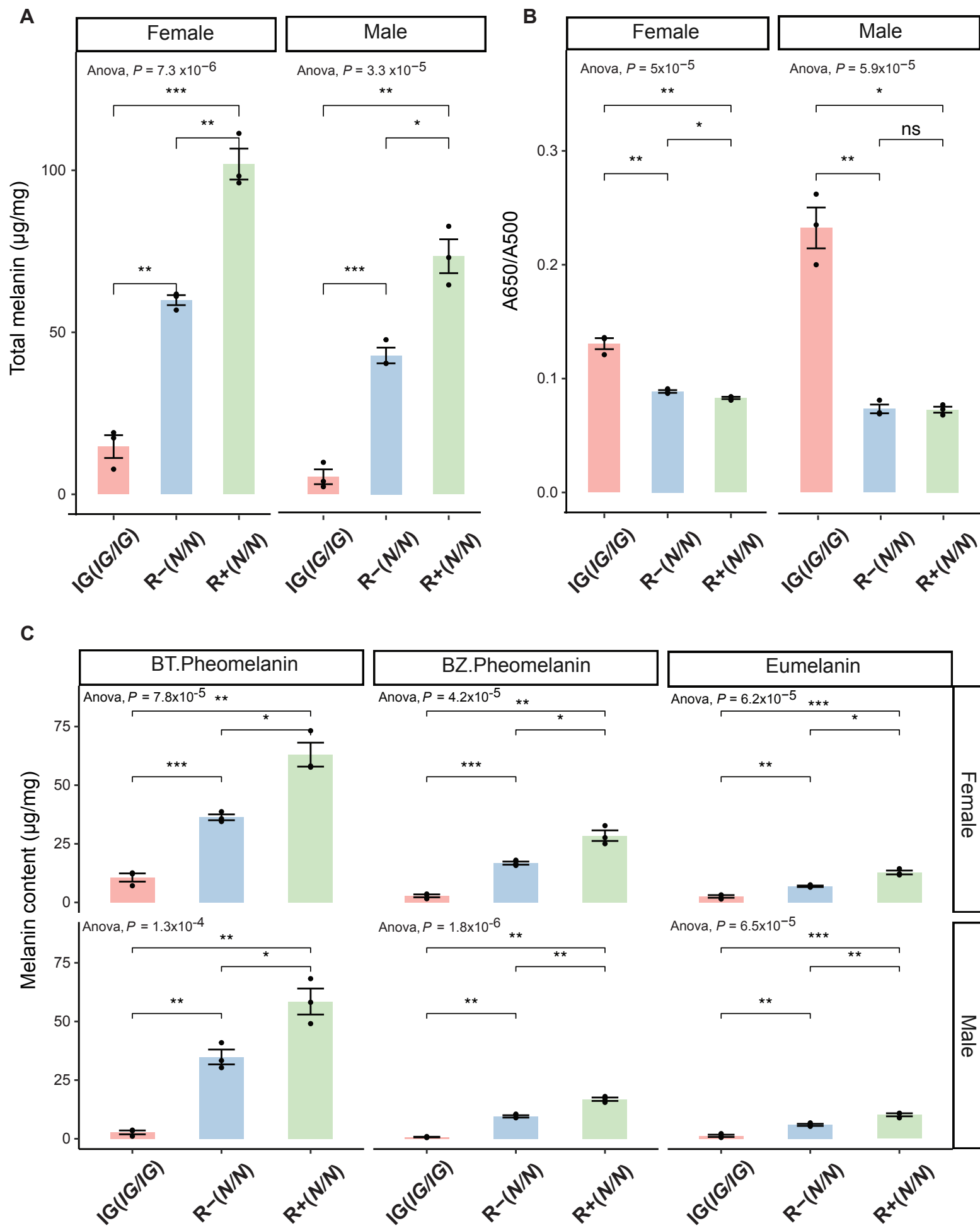

Supplement: S1 Fig — (A) Levels of total melanin in wild-type birds (R+ and R-) and IG birds analyzed by Soluene-350 solubilization. (B) A650/A500 ratios analyzed by Soluene-350 solubilization. (C), eumelanin (EM), benzothiazine-pheomelanin (BT-PM), and benzothiazole-pheomelanin (BZ-PM) analyzed as PTCA, 4-AHP, and TTCA, respectively. Feather samples were obtained from neck regions from 3 males and 3 females. Results are shown with the means ± SEM of 3 birds. ns: not significant, P > 0.05; *: P < 0.05; **: P <0.01; ***: P <0.001 (Student’s t test). (PDF) [file pgen.1010724.s001.pdf]

# HA-COMTD1

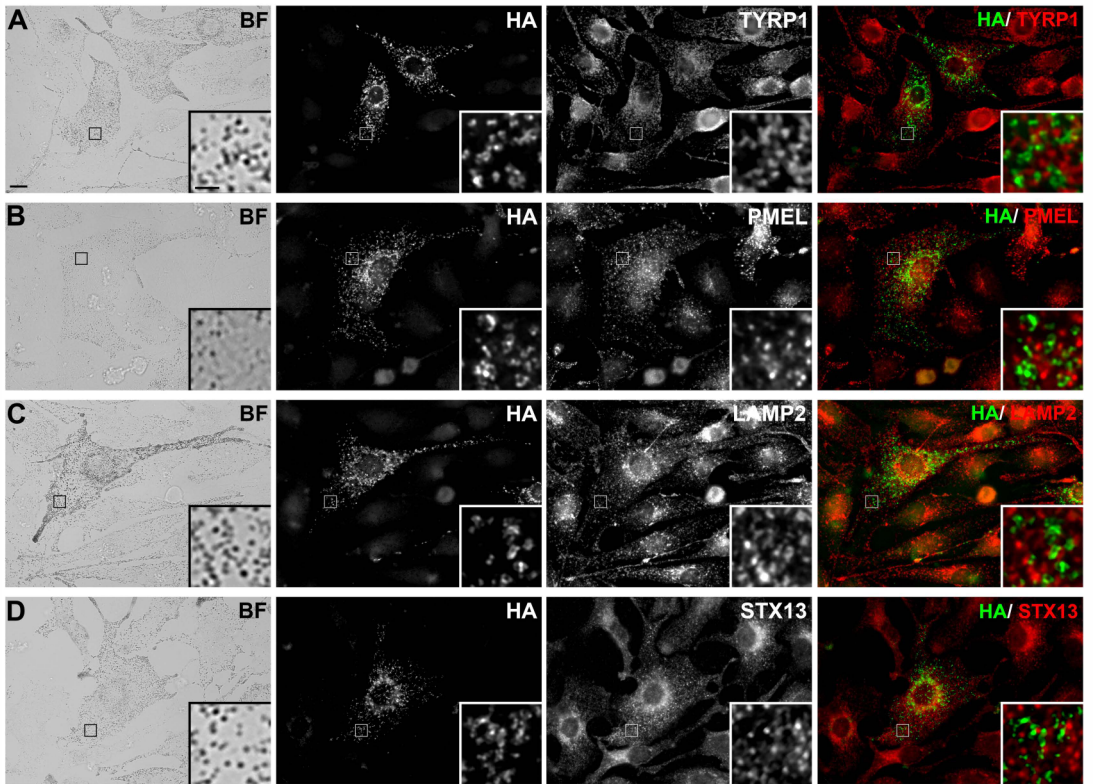

# COMTD1-HA

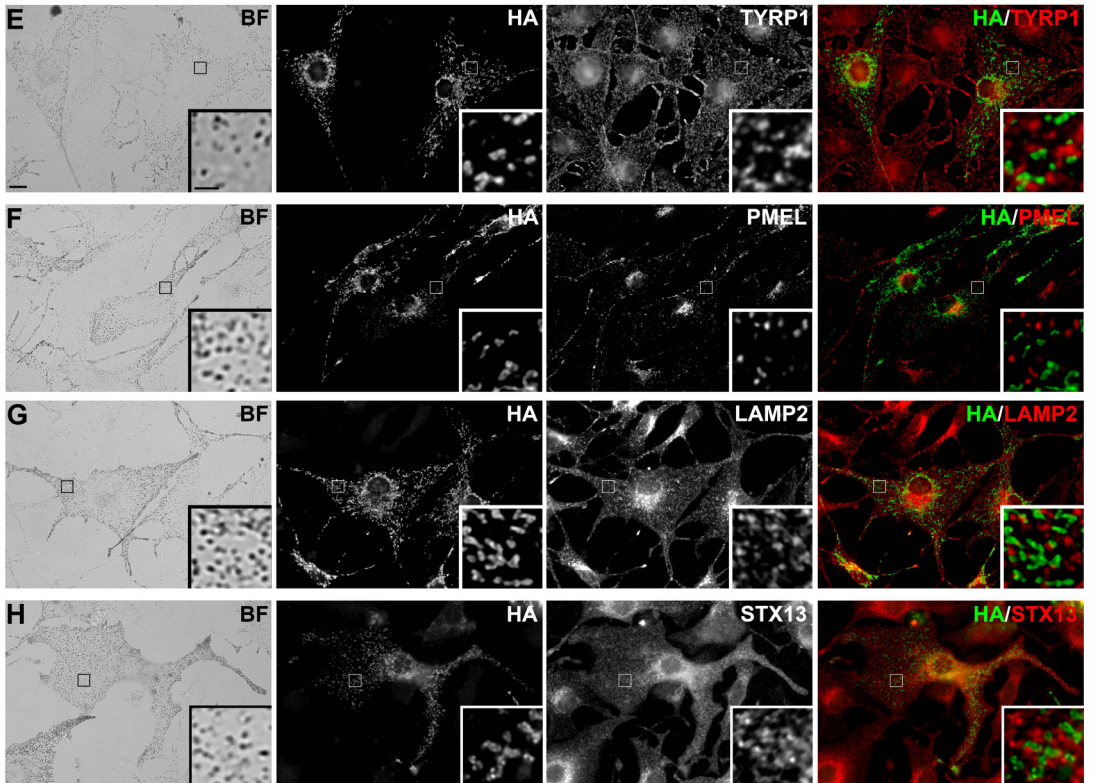

Supplement: S2 Fig — Immortalized melan-Ink4a cells were transiently transfected to express COMTD1 fused with the HA11 epitope at the N-terminus (HA-COMTD1) (A-D) or the HA11 epitope at the C-terminus (COMTD1-HA) (E-H). Two days later, cells were fixed and analyzed by bright field (BF) and immunofluorescence microscopy for HA and markers of either mature melanosomes (TYRP1; A, E), early stage melanosomes (PMEL; B, F), late endosomes/ lysosomes (LAMP2 C, G), or early endosomes (STX13; D, H). Individual images of labeled cells or the bright field image are shown in addition to an overlay of HA (green) with the indicated marker (red). Insets show a 7-fold magnified image of the boxed region to emphasize the lack of overlap. Main scale bar, 10 μm; inset scale bar, 2 μm. (PDF) [file pgen.1010724.s002.pdf]

**A**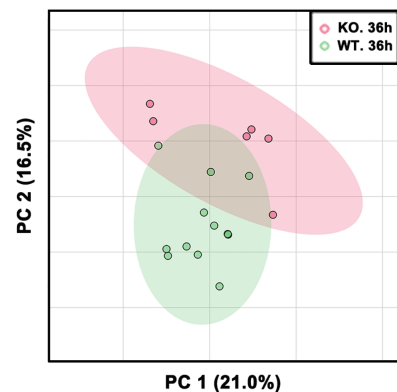**B**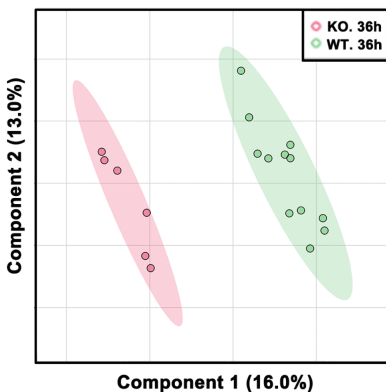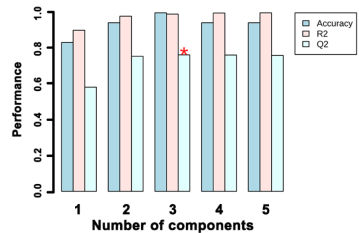**C**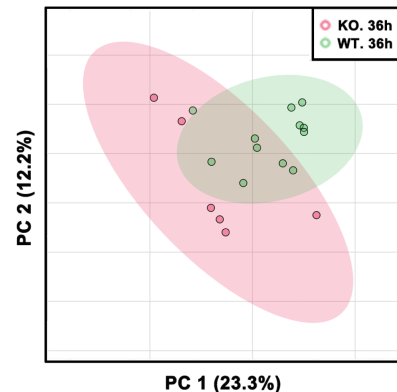**D**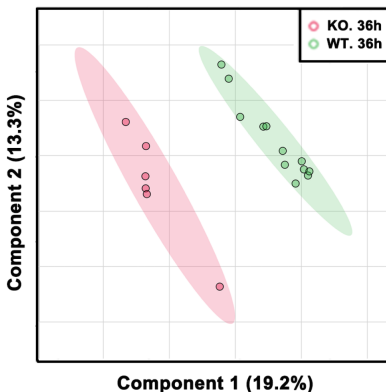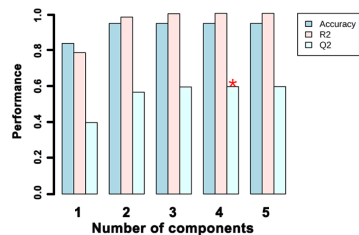**E**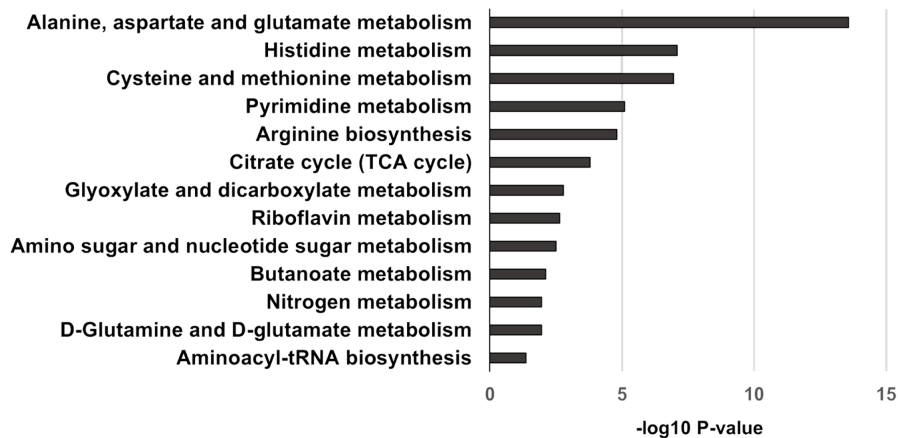

Supplement: S3 Fig — Principal component analysis (PCA) and Partial Least-Squares Discriminant Analysis (PLS-DA) with model validation results. PCA is an unsupervised multivariate analysis, which provides an unbiased overview of the metabolite features due to unawareness of the two groups compared (WT and KO in this case). PLS-DA is a multivariate analysis which considers data from the two groups and select the most discriminating metabolites that separates the two groups. This is the reason why PLS-DA show a better separation between WT and KO groups. (A) PCA in MS positive mode detection. (B) PLS-DA in MS positive mode detection. (C) PCA in MS negative mode detection. (D) PLS-DA in MS negative mode detection. (E) Significantly altered pathways impacted by Comtd1 knockout. (PDF) [file pgen.1010724.s003.pdf]
