# Supplementary material for: A frame-shift mutation in COMTD1 is associated with impaired pheomelanin pigmentation in chicken
Source: PLoS Genet. 2023 Apr 17;19(4):e1010724. doi: 10.1371/journal.pgen.1010724 (PMC10138217; doi:10.1371/journal.pgen.1010724)
Supplement: S1 Table — (DOCX) [file pgen.1010724.s004.docx]

**S1 Table. Sequence variants (non-reference alleles) in the coding region of *LRMDA*, *ZNF503*, and *COMTD1* detected by sequencing one homozygous *IG/IG* bird and the corresponding allele frequencies among non-IG *(N/-*) birds**^1^.

| Position (GalGal6) | Reference | Non-Reference | Gene | Annotation | Allele frequency of non-reference in *N/-* birds |
| --- | --- | --- | --- | --- | --- |
| 14758406 | CT | C | *LRMDA* | 3'UTR | 0.62 |
| 14758428 | T | C | *LRMDA* | 3'UTR | 0.09 |
| 14758541 | C | T | *LRMDA* | 3'UTR | 0.64 |
| 14758564 | C | G | *LRMDA* | 3'UTR | 0.10 |
| 14758714 | C | T | *LRMDA* | 3'UTR | 0.10 |
| 14758856 | A | AT | *LRMDA* | 3'UTR | 0.63 |
| 14758878 | T | G | *LRMDA* | 3'UTR | 0.11 |
| 14759095 | T | C | *LRMDA* | 3'UTR | 0.87 |
| 14759275 | T | C | *LRMDA* | 3'UTR | 0.82 |
| 14759560 | GA | G | *LRMDA* | 3'UTR | 0.42 |
| 14861168 | T | A | *LRMDA* | synonymous | 0.09^2^ |
| 15419678 | G | T | *ZNF503* | 3'UTR | 0.34 |
| 15419735 | A | AAAAC-AAAC | *ZNF503* | 3'UTR | 0.19 |
| 15419759 | C | A | *ZNF503* | 3'UTR | 0.81 |
| 15391857 | A | G | *COMTD1* | 5'UTR | 0.55 |
| 15392022 | A | T | *COMTD1* | 5'UTR | 0.92 |
| 15392096 | C | T | *COMTD1* | 5'UTR | 0.48 |
| 15671917 | T | C | *COMTD1* | 5'UTR | 0.93 |
| 15671970 | G | A | *COMTD1* | synonymous | 0.94 |
| 15672179 | C | T | *COMTD1* | non-synonymous, V77A | 0.91 |
| 15675521 | T | TCT | *COMTD1* | frame-shift | 0.02^2^ |
| 15678700 | A | G | *COMTD1* | 3'UTR | 0.67 |
| 15679829 | GA | G | *COMTD1* | 3'UTR | 0.50 |

^1^ The frequencies of non-reference alleles are based on 46 non-IG birds (*N*/-), which include 45 samples with “Red” phenotype listed in **S3 Table**, plus whole genome sequencing data for one non-IG homozygote (*N*/*N*) from our experimental pedigree.

^2^ No non-reference homozygote presents among the 46 non-IG (*N*/-) samples.
