# Supplementary material for: A frame-shift mutation in COMTD1 is associated with impaired pheomelanin pigmentation in chicken
Source: PLoS Genet. 2023 Apr 17;19(4):e1010724. doi: 10.1371/journal.pgen.1010724 (PMC10138217; doi:10.1371/journal.pgen.1010724)
Supplement: S2 Table — (DOCX) [file pgen.1010724.s005.docx]

**S2 Table. The pheomelanic phenotype distribution in a White Leghorn (OS-line) X red junglefowl pedigree material according to genotype of the 2-bp-insertion (*TC*) in *COMTD1.***

|  | ***COMTD1* genotype^1^** | |  |
| --- | --- | --- | --- |
| **Phenotype** | *CT/CT* | *WT/–* | Total |
| No Red^2^ | 25 | 24 | 49 |
| Red | 0 | 58 | 58 |
| Total | 25 | 82 | 107 |

^1^Alleles: *CT*=2-bp-insertion; *WT*=wild-type, no insertion; *–* = *WT* or *CT*

^2^Animals could still have eumelanic pigmentation.
