# Supplementary material for: A frame-shift mutation in COMTD1 is associated with impaired pheomelanin pigmentation in chicken
Source: PLoS Genet. 2023 Apr 17;19(4):e1010724. doi: 10.1371/journal.pgen.1010724 (PMC10138217; doi:10.1371/journal.pgen.1010724)
Supplement: S3 Table — Results deduced from whole genome sequencing data from the individuals listed in S4 Table. (DOCX) [file pgen.1010724.s006.docx]

**S3 Table. Genotype distribution of the 2-bp-insertion in *COMTD1* associated with the Inhibitor of Gold (IG) phenotype in different populations sorted by phenotype. Results deduced from whole genome sequencing data from the individuals listed in S4 Table.**

|  |  |  |  | **Genotype** |  |
| --- | --- | --- | --- | --- | --- |
| **Breed** | Phenotype | n | *CT/CT* | *WT/CT* | *WT/WT* |
| **Beijing You** | Red | 1 |  |  | 1 |
| **Black breasted Toutenkou** | Red | 1 |  |  | 1 |
| **Black tailed buff Japanese Bantam** | Red | 1 |  |  | 1 |
| **Brown line** | Red | 1 |  |  | 1 |
| **Buff Orpington** | Red | 1 |  |  | 1 |
| **Buttercup** | Red | 1 |  |  | 1 |
| **Chahua** | Red | 1 |  |  | 1 |
| **Dark brown Leghorn** | Red | 1 |  |  | 1 |
| **Gold Brahma** | Red | 1 |  |  | 1 |
| **Huiyang Bearded** | Red | 1 |  |  | 1 |
| **Miyi fowl** | Red | 4 |  |  | 4 |
| **Mottled Aseel** | Red | 1 |  |  | 1 |
| **Pengxian yellow fowl** | Red | 6 |  |  | 6 |
| **Porcelain Booted bantam** | Red | 1 |  | 1 |  |
| **red junglefowl** | Red | 15 |  | 1 | 14 |
| **Rhode Island** | Red | 2 |  |  | 2 |
| **Roman commercial layer** | Red | 1 |  |  | 1 |
| **Shimian caoke fowl** | Red | 3 |  |  | 3 |
| **Smyth line** | Red | 1 |  |  | 1 |
| **Spangled Orloff** | Red | 1 |  |  | 1 |
| **Fayoumi** | Barred | 1 |  |  | 1 |
| **Emei black fowl** | Black | 5 |  |  | 5 |
| **Java** | Black | 2 | 1 |  | 1 |
| **Java** | Auburn | 2 |  |  | 2 |
| **Java** | Black mottled | 3 |  |  | 3 |
| **Java** | White | 2 |  |  | 2 |
| **Jiuyuan black-bone fowl** | Black | 4 |  |  | 4 |
| **Kedu Hitam** | Black | 10 |  |  | 10 |
| **Langshan** | Black | 1 |  |  | 1 |
| **Mottled Houdan** | Black | 1 |  | 1 |  |
| **Mottled Japanese Bantam** | Black | 2 |  |  | 2 |
| **Muchuan black-bone fowl** | Black | 5 |  | 1 | 4 |
| **Rosecomb Bantam** | Black | 1 |  |  | 1 |
| **Rumpless Araucana** | Black | 1 |  |  | 1 |
| **Shamo** | Black | 1 |  |  | 1 |
| **Shouguang** | Black | 1 |  |  | 1 |
| **Sumatra** | Black | 10 |  | 2 | 8 |
| **Tianfu black-bone fowl** | Black | 4 |  |  | 4 |
| **White crested Polish** | Black | 1 |  | 1 |  |
| **Sundheimer** | Light | 1 |  | 1 |  |
| **Booted Bantam** | Millefleur | 2 |  |  | 2 |
| **German Faverolles** | Salmon | 1 |  |  | 1 |
| **Sebright Bantam** | Silver | 1 | 1 |  |  |
| **East Friesian Gulls** | Silver penciled | 1 |  | 1 |  |
| **Broiler lines** | White | 21 |  | 5 | 16 |
| **Jinyang silky fowl** | White | 6 |  |  | 6 |
| **Leghorn** | White | 29 | 24 | 5 |  |
| **Lhasa** | White | 4 | 1 | 2 | 1 |
| **Plymouth Rock** | White | 1 |  |  | 1 |
| **Rhode Island** | White | 2 |  |  | 2 |
| **Silkies** | White | 1 |  |  | 1 |
