# Supplementary material for: A frame-shift mutation in COMTD1 is associated with impaired pheomelanin pigmentation in chicken
Source: PLoS Genet. 2023 Apr 17;19(4):e1010724. doi: 10.1371/journal.pgen.1010724 (PMC10138217; doi:10.1371/journal.pgen.1010724)
Supplement: S4 Table — (DOCX) [file pgen.1010724.s007.docx]

**S4 Table. Public whole genome sequence data from chicken used in this study.**

| **Breed** | Variety | Library Type | BioSample accession no | *COMTD1* genotype |
| --- | --- | --- | --- | --- |
| **Aseel** | Red Mottled | Pool | SAMEA104432184 | *WT/WT* |
| **Beijing You** | Red | Individual | SAMN02486156 | *WT/WT* |
| **Booted Bantam** | Millefleur | Individual | SAMEA6529990 | *WT/WT* |
| **Booted Bantam** | Millefleur | Pool | SAMEA104432193 | *WT/WT* |
| **Booted bantam** | Red Porcelain | Individual | SAMEA5930879 | *WT/CT* |
| **Brahma** | Gold | Pool | SAMEA104432186 | *WT/WT* |
| **Broiler** | White | Individual | SAMN07327526 | *WT/CT* |
| **Broiler** | White | Individual | SAMN07327530 | *WT/CT* |
| **Broiler** | White | Individual | SAMN07327527 | *WT/WT* |
| **Broiler** | White | Individual | SAMN07327528 | *WT/WT* |
| **Broiler** | White | Individual | SAMN07327529 | *WT/WT* |
| **Brown Line** | Red | Individual | SAMN02941161 | *WT/WT* |
| **Burmese RJF** | Red | Pool | SAMEA104432195 | *WT/WT* |
| **Chahua** | Red | Pool | SAMN04455019 | *WT/WT* |
| **Commercial Layer** | Roman | Individual | SAMN02712022 | *WT/WT* |
| **East Friesian Gulls** | Silver Penciled | Pool | SAMEA104432208 | *WT/CT* |
| **Emei black fowl** | Black | Individual | SAMN04349707 | *WT/WT* |
| **Emei black fowl** | Black | Individual | SAMN04349707 | *WT/WT* |
| **Emei black fowl** | Black | Individual | SAMN04349707 | *WT/WT* |
| **Emei black fowl** | Black | Individual | SAMN04349707 | *WT/WT* |
| **Emei black fowl** | Black | Individual | SAMN04349707 | *WT/WT* |
| **Fat Line** | White | Individual | SAMN02782189 | *WT/CT* |
| **Fat Line** | White | Individual | SAMN02782192 | *WT/CT* |
| **Fat Line** | White | Individual | SAMN03009569 | *WT/CT* |
| **Fat Line** | White | Individual | SAMN02782188 | *WT/WT* |
| **Fat Line** | White | Individual | SAMN02782190 | *WT/WT* |
| **Fat Line** | White | Individual | SAMN02782191 | *WT/WT* |
| **Fat Line** | White | Individual | SAMN02782193 | *WT/WT* |
| **Fat Line** | White | Individual | SAMN02782194 | *WT/WT* |
| **Fat Line** | White | Individual | SAMN02782195 | *WT/WT* |
| **Fat Line** | White | Individual | SAMN03009570 | *WT/WT* |
| **Fat Line** | White | Individual | SAMN03009571 | *WT/WT* |
| **Fat Line** | White | Individual | SAMN03009572 | *WT/WT* |
| **Fat Line** | White | Individual | SAMN03009573 | *WT/WT* |
| **Fat Line** | White | Individual | SAMN03009574 | *WT/WT* |
| **Fat Line** | White | Individual | SAMN03009575 | *WT/WT* |
| **Fat Line** | White | Individual | SAMN03009576 | *WT/WT* |
| **Fayoumi** | Barred | Pool | SAMN06612097 | *WT/WT* |
| **German Faverolles** | Salmon | Pool | SAMEA104432192 | *WT/WT* |
| **Houdan** | Mottled | Pool | SAMN13810356 | *WT/CT* |
| **Huiyang** | Bearded | Pool | SAMN04364307 | *WT/WT* |
| **Indochinese RJF** | Red | Pool | SAMEA104432194 | *WT/WT* |
| **Japanese Bantam** | Black Mottled | Individual | SAMEA6529984 | *WT/WT* |
| **Japanese Bantam** | Black Mottled | Pool | SAMEA104432190 | *WT/WT* |
| **Japanese Bantam** | Black Tailed Buff | Pool | SAMEA104432188 | *WT/WT* |
| **Java** | Black | Individual | SAMD00077887 | *CT/CT* |
| **Java** | Auburn | Individual | SAMD00077882 | *WT/WT* |
| **Java** | Auburn | Individual | SAMD00077883 | *WT/WT* |
| **Java** | Black mottled | Individual | SAMD00077884 | *WT/WT* |
| **Java** | White | Individual | SAMD00077885 | *WT/WT* |
| **Java** | Black mottled | Individual | SAMD00077886 | *WT/WT* |
| **Java** | Black | Individual | SAMD00077888 | *WT/WT* |
| **Java** | White | Individual | SAMD00077890 | *WT/WT* |
| **Java** | Black mottled | Individual | SAMD00077891 | *WT/WT* |
| **Jinyang silky fowl** | White | Individual | SAMN04349707 | *WT/WT* |
| **Jinyang silky fowl** | White | Individual | SAMN04349707 | *WT/WT* |
| **Jinyang silky fowl** | White | Individual | SAMN04349707 | *WT/WT* |
| **Jinyang silky fowl** | White | Individual | SAMN04349707 | *WT/WT* |
| **Jinyang silky fowl** | White | Individual | SAMN04349707 | *WT/WT* |
| **Jinyang silky fowl** | White | Individual | SAMN04349707 | *WT/WT* |
| **Jiuyuan black-bone fowl** | Black | Individual | SAMN04349707 | *WT/WT* |
| **Jiuyuan black-bone fowl** | Black | Individual | SAMN04349707 | *WT/WT* |
| **Jiuyuan black-bone fowl** | Black | Individual | SAMN04349707 | *WT/WT* |
| **Jiuyuan black-bone fowl** | Black | Individual | SAMN04349707 | *WT/WT* |
| **Kedu Hitam** | Black | Individual | SAMD00077872 | *WT/WT* |
| **Kedu Hitam** | Black | Individual | SAMD00077873 | *WT/WT* |
| **Kedu Hitam** | Black | Individual | SAMD00077874 | *WT/WT* |
| **Kedu Hitam** | Black | Individual | SAMD00077875 | *WT/WT* |
| **Kedu Hitam** | Black | Individual | SAMD00077876 | *WT/WT* |
| **Kedu Hitam** | Black | Individual | SAMD00077877 | *WT/WT* |
| **Kedu Hitam** | Black | Individual | SAMD00077878 | *WT/WT* |
| **Kedu Hitam** | Black | Individual | SAMD00077879 | *WT/WT* |
| **Kedu Hitam** | Black | Individual | SAMD00077880 | *WT/WT* |
| **Kedu Hitam** | Black | Individual | SAMD00077881 | *WT/WT* |
| **Langshan** | Black | Pool | SAMN13810355 | *WT/WT* |
| **Leghorn** | Dark Brown | Individual | SAMN03177330 | *WT/WT* |
| **Leghorn** | White | Individual | SAMD00077893 | *CT/CT* |
| **Leghorn** | White | Individual | SAMD00077894 | *CT/CT* |
| **Leghorn** | White | Individual | SAMEA104432172 | *CT/CT* |
| **Leghorn** | White | Individual | SAMEA104432173 | *CT/CT* |
| **Leghorn** | White | Individual | SAMEA104432174 | *CT/CT* |
| **Leghorn** | White | Individual | SAMEA104432178 | *CT/CT* |
| **Leghorn** | White | Individual | SAMEA104432179 | *CT/CT* |
| **Leghorn** | White | Individual | SAMEA104432180 | *CT/CT* |
| **Leghorn** | White | Individual | SAMEA1069020 | *CT/CT* |
| **Leghorn** | White | Individual | SAMN02486166 | *CT/CT* |
| **Leghorn** | White | Individual | SAMN03438107 | *CT/CT* |
| **Leghorn** | White | Individual | SAMN03438108 | *CT/CT* |
| **Leghorn** | White | Individual | SAMN03459116 | *CT/CT* |
| **Leghorn** | White | Individual | SAMN03459118 | *CT/CT* |
| **Leghorn** | White | Individual | SAMN03459119 | *CT/CT* |
| **Leghorn** | White | Individual | SAMN03459120 | *CT/CT* |
| **Leghorn** | White | Individual | SAMN03940091 | *CT/CT* |
| **Leghorn** | White | Individual | SAMN03940092 | *CT/CT* |
| **Leghorn** | White | Individual | SAMN03940093 | *CT/CT* |
| **Leghorn** | White | Individual | SAMN07344373 | *CT/CT* |
| **Leghorn** | White | Pool | SAMN06110480 | *CT/CT* |
| **Leghorn** | White | Pool | SAMN06110480 | *CT/CT* |
| **Leghorn** | White | Pool | SAMN06110480 | *CT/CT* |
| **Leghorn** | White | Pool | SAMN06110480 | *CT/CT* |
| **Leghorn** | White | Individual | SAMD00077892 | *WT/CT* |
| **Leghorn** | White | Individual | SAMEA104432175 | *WT/CT* |
| **Leghorn** | White | Individual | SAMEA104432176 | *WT/CT* |
| **Leghorn** | White | Individual | SAMN07344374 | *WT/CT* |
| **Leghorn** | White | Individual | SAMN07344375 | *WT/CT* |
| **Lhasa** | White | Pool | SAMN04455079 | *CT/CT* |
| **Lhasa** | White | Pool | SAMN04455031 | *WT/CT* |
| **Lhasa** | White | Pool | SAMN04455095 | *WT/CT* |
| **Lhasa** | White | Pool | SAMN04455035 | *WT/WT* |
| **Miyi fowl** | Red | Individual | SAMN04349707 | *WT/WT* |
| **Miyi fowl** | Red | Individual | SAMN04349707 | *WT/WT* |
| **Miyi fowl** | Red | Individual | SAMN04349707 | *WT/WT* |
| **Miyi fowl** | Red | Individual | SAMN04349707 | *WT/WT* |
| **Muchuan black-bone fowl** | Black | Individual | SAMN04349707 | *WT/CT* |
| **Muchuan black-bone fowl** | Black | Individual | SAMN04349707 | *WT/WT* |
| **Muchuan black-bone fowl** | Black | Individual | SAMN04349707 | *WT/WT* |
| **Muchuan black-bone fowl** | Black | Individual | SAMN04349707 | *WT/WT* |
| **Muchuan black-bone fowl** | Black | Individual | SAMN04349707 | *WT/WT* |
| **Orloff** | Red spangled | Individual | SAMEA6529962 | *WT/WT* |
| **Orpington** | Buff | Pool | SAMEA104432209 | *WT/WT* |
| **Pengxian yellow fowl** | Red | Individual | SAMN04349707 | *WT/WT* |
| **Pengxian yellow fowl** | Red | Individual | SAMN04349707 | *WT/WT* |
| **Pengxian yellow fowl** | Red | Individual | SAMN04349707 | *WT/WT* |
| **Pengxian yellow fowl** | Red | Individual | SAMN04349707 | *WT/WT* |
| **Pengxian yellow fowl** | Red | Individual | SAMN04349707 | *WT/WT* |
| **Pengxian yellow fowl** | Red | Individual | SAMN04349707 | *WT/WT* |
| **Plymouth Rock** | White | Individual | SAMN02486167 | *WT/WT* |
| **Polish** | White Crested Black | Individual | SAMN03177328 | *WT/CT* |
| **Red Junglefowl** | Java | Individual | SAMD00077852 | *WT/WT* |
| **Red Junglefowl** | Java | Individual | SAMD00077853 | *WT/WT* |
| **Red Junglefowl** | Java | Individual | SAMD00077854 | *WT/WT* |
| **Red Junglefowl** | Sumatra | Individual | SAMD00077855 | *WT/WT* |
| **Red Junglefowl** | Sumatra | Individual | SAMD00077856 | *WT/WT* |
| **Red Junglefowl** | Red | Individual | SAMN02333832 | *WT/CT* |
| **Red Junglefowl** | Red | Individual | SAMN02333833 | *WT/WT* |
| **Red Junglefowl** | Red | Individual | SAMN02486161 | *WT/WT* |
| **Red Junglefowl** | Red | Individual | SAMN02712039 | *WT/WT* |
| **Red Junglefowl** | Red | Individual | SAMN02712040 | *WT/WT* |
| **Red Junglefowl** | Red | Individual | SAMN02712041 | *WT/WT* |
| **Red Junglefowl** | Red | Individual | SAMN02712042 | *WT/WT* |
| **Red Junglefowl** | Red | Individual | SAMN02712043 | *WT/WT* |
| **Rhode Island** | Red | Individual | SAMN02486160 | *WT/WT* |
| **Rhode Island** | Red | Pool | SAMEA104432211 | *WT/WT* |
| **Rhode Island** | White | Individual | SAMN02444485 | *WT/WT* |
| **Rhode Island** | White | Individual | SAMN02444503 | *WT/WT* |
| **Rose Comb Bantam** | Black | Pool | SAMEA104432185 | *WT/WT* |
| **Rumpless Araucana** | Black | Pool | SAMEA104432183 | *WT/WT* |
| **Sebright Bantam** | Silver | Pool | SAMEA104432213 | *CT/CT* |
| **Shamo** | Black | Pool | SAMEA104432215 | *WT/WT* |
| **Shimian caoke fowl** | Red head black | Individual | SAMN04349707 | *WT/WT* |
| **Shimian caoke fowl** | Red head black | Individual | SAMN04349707 | *WT/WT* |
| **Shimian caoke fowl** | Red head black | Individual | SAMN04349707 | *WT/WT* |
| **Shouguang** | Black | Individual | SAMN02486162 | *WT/WT* |
| **Sicilian Buttercup** | Red | Individual | SAMN03177329 | *WT/WT* |
| **Silkie** | White | Pool | SAMEA104432214 | *WT/WT* |
| **Smyth Line** | Red | Individual | SAMN02941162 | *WT/WT* |
| **Sumatra** | Black | Individual | SAMD00077868 | *WT/CT* |
| **Sumatra** | Black | Pool | SAMEA104432212 | *WT/CT* |
| **Sumatra** | Black | Individual | SAMD00077862 | *WT/WT* |
| **Sumatra** | Black | Individual | SAMD00077864 | *WT/WT* |
| **Sumatra** | Black | Individual | SAMD00077865 | *WT/WT* |
| **Sumatra** | Black | Individual | SAMD00077866 | *WT/WT* |
| **Sumatra** | Black | Individual | SAMD00077867 | *WT/WT* |
| **Sumatra** | Black | Individual | SAMD00077869 | *WT/WT* |
| **Sumatra** | Black | Individual | SAMD00077870 | *WT/WT* |
| **Sumatra** | Black | Individual | SAMD00077871 | *WT/WT* |
| **Sundheimer** | Light | Pool | SAMEA104432216 | *WT/CT* |
| **Tianfu black-bone fowl** | Black | Individual | SAMN04349707 | *WT/WT* |
| **Tianfu black-bone fowl** | Black | Individual | SAMN04349707 | *WT/WT* |
| **Tianfu black-bone fowl** | Black | Individual | SAMN04349707 | *WT/WT* |
| **Tianfu black-bone fowl** | Black | Individual | SAMN04349707 | *WT/WT* |
| **Toutenkou** | Black breasted red | Pool | SAMEA104432217 | *WT/WT* |
