# Supplementary material for: A frame-shift mutation in COMTD1 is associated with impaired pheomelanin pigmentation in chicken
Source: PLoS Genet. 2023 Apr 17;19(4):e1010724. doi: 10.1371/journal.pgen.1010724 (PMC10138217; doi:10.1371/journal.pgen.1010724)
Supplement: S5 Table — (DOCX) [file pgen.1010724.s008.docx]

**S5 Table. Detailed data for metabolic changes in the TCA cycle, the GSSG-GSH balance, and cysteine and methionine metabolism.**

| **TCA cycle** | | | | | | |
| --- | --- | --- | --- | --- | --- | --- |
| **Name** | **Fold change** | **Up/down** | **P-value** | ***m/z***^1^ | **rt (min)**^2^ |  |
| Succinate [M+H]^+^ | 2.55 | Up | 0.0006 | 119.0349 | 7.39 |  |
| Citrate [M+Na] ^+^ | 1.47 | Down | 0.0008 | 215.0155 | 3.23 |  |
| Malate [M+Na] ^+^ | 1.59 | Down | 0.001 | 157.0099 | 1.93 |  |
| Isocitrate [M+Na] ^+^ | 1.51 | Down | 0.01 | 215.0144 | 1.98 |  |
| Fumarate [M-H]^-^ | 1.34 | Down | 0.03 | 115.0016 | 3.67 |  |
| Pyruvate [M+HCOONa] ^+^ | 1.46 | Down | 0.0003 | 170.0413 | 2.07 |  |
|  | | | | | | |
| **GSSG - GSH balance** | | | | | | |
| **Name** | **Fold change** | **Up/down** | **P-value** | ***m/z***^1^ | **rt (min)**^2^ |  |
| Glutamine [M-H]^-^ | 1.93 | Up | 0.006 | 145.0598 | 1.38 |  |
| L-Glutamic acid [M+H] ^+^ | 1.35 | Up | 0.03 | 148.0601 | 1.42 |  |
| L-Glutathione [M-H]^-^ | 1.33 | Up | 0.14 | 306.0752 | 2.92 |  |
| Glutathione disulfide [M+H] ^+^ | 2.00 | Up | 0.003 | 613.1584 | 4.99 |  |
| (5-S/2-S) Cysteinyldopa [M+H] ^+^ | 1.45 | Down | 0.025 | 317.0794 | 4.14 |  |
|  | 1.73 | Down | 0.009 | 317.0797 | 1.68 |  |
|  | | | | | | |
| **Cysteine and methionine metabolism** | | | | | | |
| **Name** | **Fold change** | **Up/down** | **P-value** | ***m/z***^1^ | **rt (min)**^2^ |  |
| S-Adenosyl-methionine [M+H] ^+^ | 1.43 | Down | 0.01 | 399.1447 | 1.40 |  |
| S-Adenosyl-homocysteine [M+H] ^+^ | 5.64 | Up | 0.002 | 385.1281 | 5.11 |  |
| 5'-Methyl thioadenosine [M+H] ^+^ | 2.65 | Up | 0.0003 | 298.0967 | 7.39 |  |
| Cystathionine [M+Na] ^+^ | 1.64 | Up | 0.015 | 245.0575 | 4.98 |  |
| 2-Oxobutanoate [M+H] ^+^ | 1.16 | Down | 0.045 | 103.0385 | 1.46 |  |
| Pyruvate [M+HCOONa] ^+^ | 1.46 | Down | 0.0003 | 170.0413 | 2.07 |  |
| Homocysteine [M-H]^-^ | 1.70 | Down | 0.04 | 134.0328 | 1.36 |  |
| 3-Sulfino-L-alanine [M+H] ^+^ | 1.30 | Down | 0.008 | 154.0200 | 1.51 |  |
| Aminobutanoate [M-H]^-^ | 1.52 | Down | 0.03 | 102.0542 | 1.42 |  |
|  | | | | | | |
| **Riboflavin metabolism** | | | | | | |
| **Name** | **Fold change** | **Up/down** | **P-value** | ***m/z***^1^ | **rt (min)**^2^ |  |
| Riboflavin [M+H] ^+^ | 3.37 | Down | 1.5x10^-9^ | 377.1455 | 8.98 |  |
| Riboflavin 5'-Monophosphate [M+H] ^+^ | 4.71 | Down | 1.3x10^-8^ | 457.1119 | 8.65 |  |
| Reduced riboflavin [M+Na] ^+^ | 2.65 | Down | 0.025 | 298.0967 | 7.39 |  |
|  | | | | | | |
| **Metabolites not significantly altered** | **Fold change** | **Up/down** | **P-value** | ***m/z***^1^ | **rt (min)**^2^ |  |
| L-Methionine [M-H]^-^ | 1.13 | - | 0.5271 | 148.0465 | 6.19 |  |
| 2,3-Diketo-5-methylthiopentyl-1-phosphate [M+H] ^+^ | 1.08 | - | 0.7334 | 243.0083 | 1.27 |  |
| 3-Sulfinyl pyruvate [M+Na] ^+^ | 1.02 | - | 0.8037 | 156.9647 | 1.14 |  |
| 1,2-Dihydroxy-5-(methylthio)  pent-1-en-3-one [M+H] ^+^ | 1.11 | - | 0.4577 | 163.0414 | 1.40 |  |
| S-Adenosyl methioninamine [M-H]^-^ | 1.02 | - | 0.7523 | 355.1577 | 17.19 |  |
| γ-Glutamyl-2-aminobutyrate [M+H] ^+^ | 1.12 | - | 0.3769 | 233.1125 | 2.15 |  |
| 4-Methylthio-2-oxo  butanoic acid [M+H] ^+^ | 1.02 | - | 0.6587 | 149.0234 | 12.85 |  |

^1^m/z = mass-to-charge ratio

^2^rt = retention time
