# Supplementary material for: A frame-shift mutation in COMTD1 is associated with impaired pheomelanin pigmentation in chicken
Source: PLoS Genet. 2023 Apr 17;19(4):e1010724. doi: 10.1371/journal.pgen.1010724 (PMC10138217; doi:10.1371/journal.pgen.1010724)
Supplement: S6 Table — (DOCX) [file pgen.1010724.s009.docx]

**S6 Table. Primer sequences used in this paper.**

| **Primer name** | Sequences 5'-3' |
| --- | --- |
| **Pyrosequencing** | |
| AP3UP3_5Mb_F | AACGACCAAAGTCTAGCCACGTGTGTTT |
| AP3UP3_5Mb_R | ACCAACAAAGTTCACTGCAGAA |
| AP3DWN3.5Mb_F | AACGACAAACCAAACACTAGAACTCTACTC |
| AP3DWN3.5Mb_R | AAAGCTGTACGGGACTGGGCTGAG |
| AP3UP500Kb_F | AACGACGCAGCTACATTGAAACAAAGGAAA |
| AP3UP500Kb_R | AATGAGCAGGGTGGTGTCTGTTCT |
| APUP1500Kb_F | AACGACGCTGTGGATATGACCTCTCATT |
| APUP1500Kb_R | TGAATCAGTGCTTGCTTTGC |
| **Microsatellites** | |
| ADL142_F | CAGCCAATAGGGATAAAAGC |
| ADL142_R | CTGTAGATGCCAAGGAGTGC |
| MCW250_F | CAGAATTTAGAGACTGTCTAC |
| MCW250_R | ATACGGTAGCTCTGTTGCAAG |
| IG5_F | CACGACGTTGTAAAACGACGCAGAACAATCCCCAGAATA |
| IG5_R | CTGCAGTGTCTTGCATTTGTCACGACGTTGTAAAACGA |
| IG6_F | CAGTCAAGAAGAGAAAAACCTTCC |
| IG6_R | ATTCAGTTTTCCACCAGCAA |
| IG1250Kb_F | CACGACGTTGTAAAACGACCACACCAGCAGTTCCATCAT |
| IG1250Kb_R | GTGGCACTCAGGGTGGTAGT |
| **Resequencing** | |
| 15.66_2Mb_F | CGCAGTTCTCCTCTTTGGTC |
| 15.66_2Mb_R | ATGGTGGCACTCCTGGTAAC |
| 15.44_2Mb_F | TTAATCCCGTCCAGTTCTGC |
| 15.44_2Mb_R | AGCAGGGGCTGTGTGTGTAT |
| 15.44_3Mb_F | TGGAAATAGCCAGACAAACAAA |
| 15.44_3Mb_R | CCAGTTAGAGCAACCCAGTGA |
| 15.44Mb_F | CTGGACGCTTTAATGGATCA |
| 15.44Mb_R | TGCTTTGAATTTTGGTGATGA |
| VDAC_F | AAACACAGCCCTTCCATGTC |
| VDAC_R | GCGAGTGTGGTTGTGCATAC |
| COМTD1_F_REG | GGAAAGCCACTGTGGAAAGA |
| COМTD1_R_REG | AACAAGGCAATGGACAGGAC |
| COMTDI_F_REG2 | CACCТCCAGCTGCTGAACA |
| COМTD1_R_REG2 | GAAGTCATGCGTTAAAGTCAAGC |
| **Diagnostic test for the 2-bp insertion (Taqman)** | |
| COMTD1_F | AGGCAGGAGTAGATCATAAAATTGACCTA |
| COMTD1_R | TGGTTTTTAAGGAAGGAAAGGAAGCA |
| COМTD1_MUT | САGСТАСТСТСАААСАСТ |
| COMTD1_NORM | CAGCTACTCAAACACТ |
| **KO genotyping** | |
| COMTD1_PCR_F | CCACCGAGCCCCTGAATG |
| COMTD1_PCR_R | CTTCTGCTCCACTTCTGCCT |
| **Quantification of gene expression** | |
| Hprt_F | GATTAGCGATGATGAACCAGGTTA |
| Hprt_R | GACATCTCGAGCAAGTCTTTCAGTC |
| Comtd1_qRT-PCR_F | GCAGAAGTGGAGCAGAAGAT |
| Comtd1_qRT-PCR_R | GGCGGTACAGTTCTCTTTGT |
