# Supplementary material for: A frame-shift mutation in COMTD1 is associated with impaired pheomelanin pigmentation in chicken
Source: PLoS Genet. 2023 Apr 17;19(4):e1010724. doi: 10.1371/journal.pgen.1010724 (PMC10138217; doi:10.1371/journal.pgen.1010724)
Supplement: S7 Table — (DOCX) [file pgen.1010724.s010.docx]

**S7 Table. Protein quantification results with experimental triplicate**.

| **Cell Line** | **Q1** | **Q2** | **Q3** | **Mean** |
| --- | --- | --- | --- | --- |
| **KO01** | 0.52 | 0.52 | 0.52 | 0.52 |
| **KO02** | 0.45 | 0.45 | 0.45 | 0.45 |
| **KO03** | 0.37 | 0.37 | 0.37 | 0.37 |
| **KO04** | 0.45 | 0.45 | 0.45 | 0.45 |
| **KO05** | 0.30 | 0.30 | 0.30 | 0.30 |
| **KO06** | 0.22 | 0.22 | 0.22 | 0.22 |
| **WT01** | 0.35 | 0.35 | 0.35 | 0.35 |
| **WT02** | 0.37 | 0.37 | 0.37 | 0.37 |
| **WT03** | 0.38 | 0.38 | 0.38 | 0.38 |
| **WT04** | 0.38 | 0.38 | 0.38 | 0.38 |
| **WT05** | 0.49 | 0.49 | 0.49 | 0.49 |
| **WT06** | 0.42 | 0.42 | 0.42 | 0.42 |
| **WT07** | 0.39 | 0.39 | 0.39 | 0.39 |
| **WT08** | 0.46 | 0.46 | 0.46 | 0.46 |
| **WT09** | 0.43 | 0.43 | 0.43 | 0.43 |
| **WT10** | 0.54 | 0.54 | 0.54 | 0.54 |
| **WT11** | 0.45 | 0.45 | 0.45 | 0.45 |
| **WT12** | 0.43 | 0.43 | 0.43 | 0.43 |
